# On the Use of Entropy Issues to Evaluate and Control the Transients in Some Epidemic Models

**DOI:** 10.3390/e22050534

**Published:** 2020-05-09

**Authors:** Manuel De la Sen, Raul Nistal, Asier Ibeas, Aitor J. Garrido

**Affiliations:** 1 Institute of Research and Development of Processes IIDP, University of the Basque Country, Campus of Leioa, PO Box 48940 Leioa (Bizkaia), Spain; raul.nistal@gmail.com (R.N.); aitor.garrido@ehu.eus (A.J.G.); 2 Department of Telecommunications and Systems Engineering, Universitat Autònoma de Barcelona, 08193 Barcelona, Spain; Asier.Ibeas@uab.cat; 3 Faculty of Engineering of Bilbao, University of the Basque Country, Rafael Moreno No. 3, 48013 Bilbao, Spain

**Keywords:** Shannon entropy, epidemic model, transient behavior, vaccination and treatment intervention controls

## Abstract

This paper studies the representation of a general epidemic model by means of a first-order differential equation with a time-varying log-normal type coefficient. Then the generalization of the first-order differential system to epidemic models with more subpopulations is focused on by introducing the inter-subpopulations dynamics couplings and the control interventions information through the mentioned time-varying coefficient which drives the basic differential equation model. It is considered a relevant tool the control intervention of the infection along its transient to fight more efficiently against a potential initial exploding transmission. The study is based on the fact that the disease-free and endemic equilibrium points and their stability properties depend on the concrete parameterization while they admit a certain design monitoring by the choice of the control and treatment gains and the use of feedback information in the corresponding control interventions. Therefore, special attention is paid to the evolution transients of the infection curve, rather than to the equilibrium points, in terms of the time instants of its first relative maximum towards its previous inflection time instant. Such relevant time instants are evaluated via the calculation of an “ad hoc” Shannon’s entropy. Analytical and numerical examples are included in the study in order to evaluate the study and its conclusions.

## 1. Introduction

Some classical works by Boltzmann, Gibbs and Maxwell have defined entropy under a statistical framework. A useful entropy concept is the Shannon entropy since it is a basic tool to quantify the amount of uncertainty in many kinds of physical or biological processes [1,2,3,4,5,6]. It may be interpreted as a quantification of information loss [1,2,3,7,8,9]. On the other hand, entropy-based tools have been also proposed to evaluate the propagation of epidemics and related public control interventions (see, for instance, [10,11,12,13,14,15,16,17] and some of the references therein). There are also models whose basic framework relies on the use of entropy tools, as for instance [13,14,15,16]. It can be also pointed out that the control designs might be incorporated to some epidemic propagation and other biological problems, see, for instance, [18,19,20,21,22,23,24,25,26,27], and, in particular, for the synthesis of decentralized control in patchy (or network node-based) interlaced environments [24,27]. A typical situation is that of several towns each with its own health center, whose susceptible and infectious populations, apart from their coupled self-dynamics among their integrating subpopulations, might also mutually interact with the subpopulations of the neighboring nodes through in-coming and out-coming fluxes.

It can be pointed out that the knowledge or estimation of the transient behavior of the infection is very relevant for the hospital management of the disease since it is necessary to manage the availability of beds and other sanitary utensils and sanitary means, in general. The work by Wang et al. in [11] pays mainly attention to the description of the transient behavior of the evolution of epidemics rather than to the equilibrium states. The main purpose in that paper was to formulate the time interval occurring between the time instant of the maximum of the infection, which gives a relative maximum of the infection evolution through time (and which zeroes the first time-derivative of the infection function), and the time instant giving its previous inflection time instant. It turns out that the knowledge of the first part of the transient evolution is very relevant to fight against the initial exploding of the illness since any eventual control intervention is typically much more efficient as far as it is taken as quickly as possible. The model proposed in [11] is a time-varying differential equation of first-order describing the infectious population which is the unique explicit one in the model. It is also pointed out in that paper that the time-varying coefficient might potentially contain the supplementary environment information to make such an equation well-posed to practically describe a concrete disease evolution. An interesting point of that work is that the infection evolution is identified with a log-normal distribution whose parameterization is selected in such a way that the entropy production rate is maximized. The above proposed theoretical first-order model has been proved to be very efficient to describe the data of SARS 2003. Alternative interpretations of the entropy in terms of maximum entropy or maximum entropy rate are given, for instance, in [12,13,14] and some references therein.

This paper studies how to link the extension of the first-order differential system proposed in [11] for the study of infection propagations to epidemic models with more integrated coupled subpopulations (such as susceptible, immune, vaccinated etc.) by introducing the coupling and control information through the time-varying coefficient which drives the basic differential equation model. It is considered relevant the control of the infection along its transient to fight more efficiently against a potential initial exploding transmission. Note that the disease-free and endemic equilibrium points and their stability properties depend on the concrete parameterization while they admit a certain design monitoring by the choice of the control and treatment gains and the use of feedback information in the corresponding controls. See, for instance [19,27]. Therefore, special attention is paid to the transients of the infection curve evolution in terms of the time instants of its first relative maximum towards its previous inflection time instant since there is a certain gap in the background literature concerning the study of such transients. The ratio of such time instants is later on considered subject to some worst-case uncertainty relations via the calculation and analysis of an “ad hoc” Shannon’s entropy. Note that entropy issues have been considered in the study of biological, evolution and epidemic models by incorporating techniques of information theory. See, for instance [11,12,13,28,29,30,31,32]. It is well-known that the entropy production theorems might be classified according to a generalized sequence of stable thermodynamic states. Also, the thermodynamic equilibrium, which is characterized by the absence of gradients of state or kinematic variables, is in a state of maximum entropy and zero entropy production [33,34]. Furthermore, linear non-equilibrium processes are associated with entropy production so that the entropy concept may be also invoked in transient processes [35]. On the other hand, it may be pointed out that uncertainties can appear in the characterization of the infection evolution through time, even in deterministic models, due to parameterization uncertainties, fluxes of populations or existing uncertainties in the initial conditions. Other mathematical techniques of interest which combine analytical and numerical issues have been also been applied to the analysis and discussion of epidemic models with eventual support of mathematical techniques on homotopy analysis and distribution functions as, forinstance, the log-normal distribution [36,37]. For instance, in [38], the SIR and SIS epidemic models are solved through the homotopy analysis method. A one-parameter family of series solutions is obtained which gives a method to ensure convergent series solutions for those kinds of models. On the other hand, in [39], the analytic solutions of an SIR epidemic model are investigated in parametric form. It is also found that the generalization of a SIR model including births and mortality with vital dynamics might be reduced to an Abel-type which greatly simplify the analysis.

The paper is organized as follows: Section 2 gives an extension of the basic model of [11] to be then compared in subsequent sections with some existing models with several subpopulations. Such a model only considers the infection evolution through time and it is based on the action of two auxiliary non-negative functions which define appropriately the time-varying coefficient which defines the first-order differential equation of the infection evolution. The model includes, as particular case, that of the abovementioned reference where both such auxiliary functions are identical to the time argument. Particular choices of those functions make it possible to consider alternative effects linked to the basic model like, for instance, the influence on the infectious subpopulation of other coupled subpopulations in more general models like, for instance, the susceptible, exposed, recovered or vaccinated ones. It is also possible to include the control effects through such a varying coefficient, if any, like for instance, the vaccination and treatment controls. Some basic formal results are stated and proved mainly concerning with the first relative maxima and inflection time instants of the infection curve through time. The above two time instants are relevant to take appropriate control interventions to fight against an initially exploding infectious disease.

Section 3 links the basic model of Section 2 with some known epidemic models which integrate more subpopulations than just the infectious one, like for instance, the susceptible and recovered subpopulations, The time-varying coefficient driving the infection evolution is defined explicitly for each of the discussed epidemic models. Basically, it is taken in mind that some relevant information of higher-order differential epidemic models concerning the transient trajectory solution can be captured by a parameter-dependent and time-varying coefficient which drives a first-order differential equation to the light of the basic model of Section 2. So, the time-varying coefficient describing the infection evolution depends in those cases of the remaining subpopulations integrated in the model. The maximum and inflection time instants are characterized for some given examples involving epidemic models of several subpopulations. In particular, the last one of the discussed theoretical examples includes the effects of vaccination and treatment intervention controls generated by linear feedback of the susceptible and infectious subpopulations, respectively. Later on, Section 4 investigates the entropy associated with the infection accordingly to the generalizations of Section 2 concerning the specific structure of the time-varying coefficient describing the infection dynamics and its links with the theoretical examples discussed in Section 3. The error of the entropy related to the reference one associated with the log-normal distribution is estimated. In practice, that property can be interpreted in terms of public medical and social interventions which control the disease propagation when introducing the controls of the last example discussed in Section 3. The second part of Section 4 is devoted to linking the entropy and inflection and maximum infection time instants and their reached values of the discussed multi-population structures to their counterparts of the maximum dissipation rate being associated to the formulation of a simpler model based on the log-normal distribution and one-dimensional infection dynamics. Some numerical tests are performed for comparisons of the entropies and its width of the basic model with two of the discussed examples in the previous sections which involve the presence of more than one integrated subpopulations. Finally, conclusions end the paper.

### Notation

$$R_{+}=\left\{\;r\in R\;:\;r>0\right\};\;R_{0+}=\left\{\;r\in R\;:\;r\geq 0\right\}=R_{+}\cup \left\{0\right\}$$

$$Z_{+}=\left\{\;r\in Z\;:\;r>0\right\};\;Z_{0+}=\left\{\;r\in Z\;:\;r\geq 0\right\}=Z_{+}\cup \left\{0\right\}$$

$$\bar{n}=\left\{0,1,\cdots ,n\right\}$$

## 2. The Basic Model Description and Some Related Technical Results

Since disease propagation can be interpreted as a thermodynamic system, it can be assumed that the rate of increase or decrease is proportional to the infection at the previous day following the approach of modelling the rate of chemical reactions, [11]. Thus, assume that the infection evolution obeys the following time-varying differential equation:

(1) $$\dot{I}\left(t\right)=\alpha \left(t\right)I\left(t\right);\;I\left(0\right)=I_{0}>0$$

where $\alpha :R_{0+}\to R_{0+}$ is continuous and time differentiable on (0, +∞). The particular structure of the varying coefficient α(t) depends on the balances between the spreading mechanism and the exerted controls during the public intervention. Such a coefficient contains the available information related to the incorporation of all the control mechanisms and the coupling dynamics between the infectious populations and the remaining interacting ones such as the susceptible, immune or vaccinated ones. By taking time-derivatives with respect to time in (1), one gets:

(2) $$\begin{array}{c} \ddot{I}\left(t\right)=\dot{\alpha }\left(t\right)I\left(t\right)+\alpha \left(t\right)\dot{I}\left(t\right)\\ =\left(\dot{\alpha }\left(t\right)/\alpha \left(t\right)+\alpha \left(t\right)\right)\dot{I}\left(t\right)\\ =\left(\dot{\alpha }\left(t\right)+\alpha^{2}\left(t\right)\right)I\left(t\right);\;\dot{I}\left(0\right)=\;{\dot{I}}_{0}=\alpha \left(0\right)I_{0} \end{array}$$

It is proposed in [11] to consider two relevant time instants in the disease evolution, namely:

(1) The inflection time instant of *I*(t) which is the date in the infection evolution at which the controlling actions take effect on the evolution. Typically, this time instant is the undulation point date in the evolution of I(t), that is the zero of $\ddot{I}\left(t\right)$, provided that the first non-zero derivative of $I^{\left(n\right)}\left(t\right)=\frac{d^{n}I\left(t\right)}{dt^{n}}$, n≥3 occurs for some even n since this last condition ensures that the undulation time instant is the inflection time instant.
(2) The critical time instant at which the spread rate turns from initial growing to decrease which can be empirically attributed to the global influence of the control interventions. This time instant is a relative maximum of *I*(t) and it satisfies the constraints $\dot{I}\left(t\right)=0$ and $\ddot{I}\left(t\right)<0$ under the reasonable assumption that ${\dot{I}}_{0}>0$.

It turns out that, along the whole disease evolution, several successive inflection points and relative maxima can happen. The subsequent result which is concerned with the non-negativity, boundedness and asymptotic vanishing property of the infection as time tends to infinity and its two first- time derivatives is immediate from the above expressions (1) and (2):

**Theorem** **1.** *The following properties hold:*

(i) *The infection population and its two first-time derivatives obey the following time evolution equations:*
  (3) $$I\left(t\right)=e^{\int_{0}^{t}\alpha \left(\tau \right)d\tau }I_{0};\dot{I}\left(t\right)=\alpha \left(t\right)e^{\int_{0}^{t}\alpha \left(\tau \right)d\tau }I_{0};\ddot{I}\left(t\right)=\left(\dot{\alpha }\left(t\right)+\alpha^{2}\left(t\right)\right)e^{\int_{0}^{t}\alpha \left(\tau \right)d\tau }I_{0};\forall t\in R_{0+}$$
(ii) I(t)>0 *;* $\forall t\in R_{0+}$ *if and only if* $I_{0}\geq 0$ *; and* I(t)=0 *;* $\forall t\in R_{0+}$ *if and only if* $I_{0}=0$ *.*
(iii) *If*$+\infty >I_{0}\geq 0$*then*$I\left(t\right)\leq KI_{0}<+\infty$*;*$\forall t\in R_{0+}$*for some*$K\in R_{+}$*if and only if*$\alpha :R_{0+}\to R_{0+}$*is such that*$\int_{0}^{t}\alpha \left(\tau \right)d\tau \leq K<+\infty$*;*$\forall t\in R_{0+}$.
(iv) I(t)→0 *as* t→+∞ *for any given finite* $I_{0}$ *if and only if* $\underset{t\to +\infty }{lim}\int_{0}^{t}\alpha \left(\tau \right)d\tau =-\infty$ *.*
(v) *If* $+\infty >I_{0}\geq 0$ *and* $\int_{0}^{t}\alpha \left(\tau \right)d\tau \leq K<+\infty$ *;* $\forall t\in R_{0+}$ *for some* $K\in R_{0+}$ *then* $\left|\dot{I}\left(t\right)\right|<+\infty$ *;* $\forall t\in R_{0+}$ *if and only if, for some* $K_{1}\in R_{+}$ *,* $\left|\alpha \left(t\right)\right|\leq K_{1}<+\infty$ *;* $\forall t\in R_{0+}$ *. If* $+\infty >I_{0}\geq 0$ *then* $\left|\dot{I}\left(t\right)\right|<+\infty$ *;* $\forall t\in R_{0+}$ *if and only if* $\left|\alpha \left(t\right)\right|e^{\int_{0}^{t}\alpha \left(\tau \right)d\tau }\leq K_{2}<+\infty$ *;* $\forall t\in R_{0+}$ *, for some* $K_{2}\in R_{+}$ *provided that* α(0)<+∞ *.*
(vi) $\dot{I}\left(t\right)\to 0$ *as* t→+∞ *for any given finite* $I_{0}$ *if and only if* $\underset{t\to +\infty }{lim}\left(\alpha \left(t\right)e^{\int_{0}^{t}\alpha \left(\tau \right)d\tau }\right)=0$ *.*
*If* $\alpha :R_{0+}\to R_{0+}$ *is bounded and* I(t)→0 *as* t→+∞ *then* $\dot{I}\left(t\right)\to 0$ *as* t→+∞ *.*
(vii) *If* $+\infty >I_{0}\geq 0$ *then* $\left|\ddot{I}\left(t\right)\right|<+\infty$ *;* $\forall t\in R_{+}$ *if and only if* $\left|\dot{\alpha }\left(t\right)+\alpha^{2}\left(t\right)\right|e^{\int_{0}^{t}\alpha \left(\tau \right)d\tau }\leq K_{3}<+\infty$ *;* $\forall t\in R_{0+}$ *, for some* $K_{3}\in R_{+}$ *.* $\ddot{I}\left(t\right)\to 0$ *as* t→+∞ *for any given finite* $I_{0}$ *if and only if* $\underset{t\to +\infty }{lim}\left(\left(\dot{\alpha }\left(t\right)+\alpha^{2}\left(t\right)\right)e^{\int_{0}^{t}\alpha \left(\tau \right)d\tau }\right)=0$ *.*
  *If*$\left(\alpha +\dot{\alpha }\right):R_{0+}\to R_{0+}$*is bounded and*I(t)→0*as*t→+∞*then*$\ddot{I}\left(t\right)\to 0$*as*t→+∞.

Note that α(t) (respectively, $\alpha \left(t\right)+\dot{\alpha }\left(t\right)$) is infinity at t=0 while it is bounded for t>0, as it happens for instance with the α—function proposed in [11], then $\dot{I}\left(t\right)$ (respectively, $\ddot{I}\left(t\right)$) is still bounded under the conditions of Theorem 1 (v) (respectively, Theorem 1 (vii)) on $R_{+}$.

Note also that the vanishing infection condition of Theorem 1 typically occurs under convergence of the solution to the disease-free equilibrium point if the disease reproduction number is less than one [19,22,23,24,27,29,30,36]. However, it can happen that the infection oscillates around some stable equilibrium or that it converges to a nonzero positive constant defining the corresponding component of the endemic equilibrium steady-state as it is discussed in the next result.

**Corollary** **1.** *The following properties hold:*

(**i**) *Assume that there exists some* $C\in R_{+}$ *such that* $\int_{0}^{t}\alpha \left(\tau \right)d\tau \to C$ *as* t→+∞ *and that* $\alpha \left(t\right),\;\dot{\alpha }\left(t\right)\to 0$ *as* t→+∞ *. Then,* $I\left(t\right)\to e^{C}I_{0}$ *,* $\dot{I}\left(t\right)\to 0$ *and* $\ddot{I}\left(t\right)\to 0$ *as* t→+∞ *.*
(**ii**) *Assume that* $\int_{0}^{t}\alpha \left(\tau \right)d\tau \to C$ *as* t→+∞ *and that* $\alpha :R_{0+}\to R_{0+}$ *is uniformly continuous. Then,* α(t) →0 *,* $I\left(t\right)\to e^{C}I_{0}$ *and* $\dot{I}\left(t\right)\to 0$ *as* t→+∞ *. Assume, in addition, that* $\dot{\alpha }:R_{0+}\to R_{0+}$ *is uniformly continuous. Then* $\ddot{I}\left(t\right)\to 0$ *as* t→+∞ *.*

**Proof** **of** **Property** **(i).** Follows directly from (1)–(3). On the other hand, since $\alpha :R_{0+}\to R_{0+}$ is uniformly continuous and the limit $\underset{t\to +\infty }{lim}\int_{0}^{t}\alpha \left(\tau \right)d\tau =C$ exists and it is finite then α(t)→0 as t→+∞ (Barbalat´s Lemma) and $I\left(t\right)\to e^{C}I_{0}$ as t→+∞ from (3), $\dot{I}:R_{+}\to R_{0+}$ is bounded, since being continuous, it cannot diverge in finite time, and $\dot{I}\left(t\right)\to 0$ as t→+∞ from (1). If, furthermore, $\dot{\alpha }:R_{0+}\to R_{0+}$ is uniformly continuous and, since $\underset{t\to +\infty }{lim}\int_{0}^{t}\dot{\alpha }\left(\tau \right)d\tau =\underset{t\to +\infty }{lim}\alpha \left(t\right)-\alpha \left(0\right)=-\alpha \left(0\right)$ then $\;\dot{\alpha }\left(t\right)\to 0$ as t→+∞ (again from Barbalat´s Lemma). Since $\alpha \left(t\right),\;\dot{\alpha }\left(t\right)\to 0$ as t→+∞ then $\ddot{I}\left(t\right)\to 0$ as t→+∞ from (2). □

Let us introduce the following definitions and lemma of usefulness for the proof of the subsequent theorem [36]:

**Definition** **1.** *Let*f:R→R*be everywhere continuous and twice differentiable at*$t_{0}\in R$*. Then,*$t_{0}$*is an undulation point (or pre-inflection point) of*f*if*$\ddot{f}\left(t_{0}\right)=0$.

*Inflection points of the continuous and twice-differentiable* f:R→R *are the undulation points of the function where the curvature changes its sign, that is, points of change of local convexity to local concavity or vice-versa. They are also the isolated extrema of* $\dot{f}:R\to R$ *. A well-known technical definition and a related result on inflection points follow:*

**Definition** **2.** *Let* f:R→R *be everywhere continuous and twice differentiable at* $t_{0}\in R$ *which is an isolated extremum of* f *(that is, a local maximum or minimum, and also an undulation point of,* f *as a result).*

**Lemma** **1.** *The following properties hold:*

*(**i**)*  *Let* f:R→R *be everywhere continuous and twice differentiable at* $t_{0}\in R$ *. Then,* $t_{0}$ *is an inflection point of* f *if* $\ddot{f}\left(t+\varepsilon \right)\ddot{f}\left(t-\varepsilon \right)<0$ *for some sufficiently small* $\varepsilon \in R_{+}$ *.*
*(**ii**)*  *Let* f:R→R *be everywhere continuous and an odd number* k(≥3) *-times differentiable, within a neighborhood of* $t_{0}\in R$ *which is an undulation point of* f *satisfying* $f^{\left(j\right)}\left(t_{0}\right)=0$ *for* j=2,3,…k−1 *and* $f^{\left(k\right)}\left(t_{0}\right)\neq 0$ *. Then,* $t_{0}$ *is an inflection point of* f *.*

The subsequent result has a very technical proof leading to the basic result that the zeros at finite time instants of $\dot{I}\left(t\right)$ and $\ddot{I}\left(t\right)$ alternate if *I*(t) is sufficiently smooth and α(t) is sufficiently smooth. In order to simplify the result proof, it is assumed, with no loss in generality, that the disease dynamics (1)–(2) has no equilibrium points such that the zeros under study are isolated.

**Theorem** **2.** *Assume that the function* $\alpha :R_{0+}\to R_{0+}$ *defined by* $\alpha \left(t\right)=-\frac{cln\left(g\left(t\right)/E\right)}{h\left(t\right)}$ *, where* $c\;,\;E\in R_{+}$ *and* $g,h:R_{0+}\to R_{0+}$ *are everywhere continuous and time-differentiable such that* g(0)=0 *with* $\underset{t\to 0}{lim}\frac{ln\left(g\left(t\right)/E\right)}{h\left(t\right)}\leq -\varepsilon$ *for some* $\varepsilon \in R_{0+}$ *, and furthermore,* $\alpha :R_{0+}\to R_{0+}$ *fulfills the constraints:*

(4) $$\alpha \left(D_{i}\right)=0;\;\dot{\alpha }\left(L_{i}\right)=-\alpha^{2}\left(L_{i}\right)$$

(5) $$\frac{h\left(L_{i}\right)\dot{g}\left(L_{i}\right)-ln\left(g\left(L_{i}\right)/E\right)\dot{h}\left(L_{i}\right)g\left(L_{i}\right)}{g\left(L_{i}\right){ln}^{2}\left(g\left(L_{i}\right)/E\right)}=K>0;\;\forall L_{i}\in L_{S}\cap \left[0,\;\bar{L}\right]$$

*for any given positive real number* $\bar{L}$ *, with* $D_{i}\in D_{S}$ *and* $L_{i}\in L_{S}$ *, where* $D_{S}=\left\{D\in R_{+}\;:\;\alpha \left(D\right)=g\left(D\right)=E\right\}\subset R_{0+}$ *and* $L_{S}=\left\{L\in R_{+}\;:\;\dot{\alpha }\left(L\right)+\alpha^{2}\left(L\right)=0\right\}\subset R_{0+}$ *are assumed to be nonempty and of zero Lebesgue measure.*

*Then, the following properties hold:*

(**i**) $g\left(L_{i}\right)=E\Leftrightarrow h\left(L_{i}\right)\dot{g}\left(L_{i}\right)>0$*, equivalently,*$D_{S}\cap L_{S}=\emptyset$.
(**ii**) *(a)* $cardL_{S}=cardD_{S}+\vartheta$ *with* ϑ={0, 1} *, and (a) if* $card\left(D_{S}\right)=card\left(L_{S}\right)\leq {ℵ}_{0}$ *(with* ${ℵ}_{0}$ *denoting the infinite cardinality of denumerable sets) then* $L_{i}<D_{i}<L_{i+1}$ *;* $\forall i\in Z_{0+}$ *for any pairs* $D_{i}\;,\;D_{i+1}\in D_{S}$ *and* $L_{i}\;,\;L_{i+1}\in L_{S}$ *fulfilling* $\left(D_{i},\;D_{i+1}\right)\cap D_{S}=\emptyset$ *and* $\left(L_{i},\;L_{i+1}\right)\cap L_{S}=\emptyset$ *,*
*(b) if* $1\leq card(L_{S})=card(D_{S})-1=\ell <\infty$ *then* $L_{i}<D_{i}<L_{i+1}$ *for* $i\in \bar{\ell -1}$ *.* $\alpha :R_{0+}\to R_{0+}$ *is subject to the constraint* c=K *,* $\dot{I}\left(D_{i}\right)=\ddot{I}\left(L_{i}\right)=0$ *;* $\forall L_{i}\in L_{S}\cap \left[0,\;L\right]$ *and* $D_{i}>L_{i}$ *.*
(**iii**) $\alpha :R_{0+}\to R_{0+}$ *is subject to* $\dot{I}\left(D_{i}\right)=\ddot{I}\left(L_{i}\right)=0$ *;* $\forall D_{i}\in D_{S}\cap \left[0,\;L\right]$ *,* $\forall L_{i}\in L_{S}\cap \left[0,\;L\right]$ *and* $D_{1}>L_{1}$ *for any* $I_{0}>0$ *.*

**Proof.**  First, note that $\dot{I}\left(D\right)=\ddot{I}\left(L\right)=0$; $\forall D\in D_{S}$, $\forall L\in L_{S}$ since α(D)=0 even if I(D)≠0. On the other hand, $L_{S}$ is the set of undulation points of $I:R_{0+}\to R_{0+}$ and it is clear that $D_{S}$ is contained in the set of relative maximum and minimum points of I(t). The properties (i)–(iii) are now proved:

**Proof** **of** **Property** **(i).** It is now proved that $D_{S}$ is the set of extreme points of I(t) which is disjoint to its set of undulation points $L_{S}$. Assume, on the contrary, that there is some $D\notin D_{S}$ such that $\dot{I}\left(D\right)=0$. Then, I(D)=0 since α(D)≠0, and then the disease-free equilibrium point is reached in finite time contradicting the fact that $\alpha \left(t\right)=-\frac{cln\left(g\left(t\right)/E\right)}{h\left(t\right)}$ is only zero at finite time for a discrete set of time instants satisfying g(t)=E so that $\dot{I}\left(D\right)=0$ if and only if $D\in D_{S}$. Then, $I\left(D\right)=\dot{I}\left(D\right)=\ddot{I}\left(D\right)=0$ is a disease-free equilibrium point which is reached in finite time which contradicts the given hypothesis. So, it is easy to see that $L_{S}$ and $D_{S}$ are discrete sets of non-negative real time instants which can be strictly ordered. Note also from (1)–(2) that:

(6) $$\alpha \left(D_{i}\right)=-\frac{cln\left(g\left(D_{i}\right)/E\right)}{h\left(D_{i}\right)}=0;\;\forall D_{i}\in D_{S}$$

(7) $$\dot{\alpha }\left(L_{i}\right)=-\alpha^{2}\left(L_{i}\right);\;\forall L_{i}\in L_{S}$$

If $D_{i}\leq D<+\infty$ then $g\left(D_{i}\right)=E$ since c≠0. Also, $\alpha \left(t\right)=-\frac{cln\left(g\left(t\right)/E\right)}{h\left(t\right)}$ and, if $L_{i}\in L_{S}$ and since $h\left(L_{i}\right)>0$, one has:

(8) $$\begin{array}{c} \alpha^{2}\left(L_{i}\right)=-\dot{\alpha }\left(L_{i}\right)=\frac{d}{dt}{\left[\frac{cln\left(g\left(t\right)/E\right)}{h\left(t\right)}\right]}_{t=L_{i}}\\ =c\frac{h\left(L_{i}\right)\dot{g}\left(L_{i}\right)/g\left(L_{i}\right)-ln\left(g\left(L_{i}\right)/E\right)\dot{h}\left(L_{i}\right)}{h^{2}\left(L_{i}\right)}=c^{2}\frac{{ln}^{2}\left(g\left(L_{i}\right)/E\right)}{h^{2}\left(L_{i}\right)} \end{array}$$

(9) $$c=\frac{h\left(L_{i}\right)\dot{g}\left(L_{i}\right)-ln\left(g\left(L_{i}\right)/E\right)\dot{h}\left(L_{i}\right)g\left(L_{i}\right)}{g\left(L_{i}\right){ln}^{2}\left(g\left(L_{i}\right)/E\right)}>0$$

Now, if there is some $L_{i}\in L_{S}\cap D_{S}$, equivalently, $L_{S}\cap D_{S}\neq \emptyset$, then $g\left(L_{i}\right)=E\Leftrightarrow h\left(L_{i}\right)\dot{g}\left(L_{i}\right)\neq 0$ from (9) since c≠0 and $ln\left(g\left(L_{i}\right)/E\right)=0$ and, furthermore, one gets from (8) that $\dot{\alpha }\left(L_{i}\right)\neq 0$ since $g\left(L_{i}\right)=E$. But one also has that $\dot{\alpha }\left(L_{i}\right)=\alpha \left(L_{i}\right)=0$, since $\dot{\alpha }\left(L_{i}\right)=-\alpha^{2}\left(L_{i}\right)$; $\forall L_{i}\in L_{S}$ from the first identity of (8). Then, $0\neq \dot{\alpha }\left(L_{i}\right)=0$ is a contradiction so that $L_{i}\notin L_{S}\cap D_{S}$. Equivalently, $D_{S}\cap L_{S}=\emptyset$. Property (i) has been proved. □

**Proof** **of** **Property** **(ii).** Since $\dot{\alpha }\left(L_{i}\right)=-\alpha^{2}\left(L_{i}\right)$ then $\ddot{\alpha }\left(L_{i}\right)=-2\alpha \left(L_{i}\right)\dot{\alpha }\left(L_{i}\right)$ so that:

$$\ddot{I}\left(L_{i}\right)=\left(\dot{\alpha }\left(L_{i}\right)+\alpha^{2}\left(L_{i}\right)\right)I\left(L_{i}\right)=0$$

$$\dddot{I}\left(L_{i}\right)=\left(\ddot{\alpha }\left(L_{i}\right)+2\alpha \left(L_{i}\right)\dot{\alpha }\left(L_{i}\right)\right)I\left(L_{i}\right)+\left(\dot{\alpha }\left(L_{i}\right)+\alpha^{2}\left(L_{i}\right)\right)\dot{I}\left(L_{i}\right)$$

$$=\left(\ddot{\alpha }\left(L_{i}\right)-2\alpha^{3}\left(L_{i}\right)\right)I\left(L_{i}\right)$$

Since the zeros of α(t) and those of its first time- derivative do not coincide since $D_{S}\cap L_{S}=\emptyset$ (from Property (i)), it turns out that the two sets of respective zeros alternate if there are not two zeros of α(t) within any open time interval of two consecutive zeros of $\dot{\alpha }\left(t\right)$ or vice-versa. One proceeds by contradiction arguments by assuming two cases which are both rebutted.

*Case 1*: Assume that there are two consecutive zeros of $\dot{I}\left(t\right)$ between two consecutive zeros of $\ddot{I}\left(t\right)$, then satisfying the constraint $0\leq L_{i}<D_{i}<D_{i+1}<L_{i+1}$ for some two consecutive time instants $D_{i}\;,D_{i+1}$ in $D_{S}$ and two consecutive time instants $L_{i}\;,L_{i+1}$ in $L_{S}$ so that $\alpha \left(D_{i}\right)=\alpha \left(D_{i+1}\right)=\ddot{I}\left(L_{i}\right)=\ddot{I}\left(L_{i+1}\right)=0$. Assume that I(t)=0 for some $t\in \left(D_{i},D_{i+1}\right)$ then $\dot{I}\left(t\right)=\alpha \left(t\right)I\left(t\right)=0$ so that $t\in D_{S}$ and then $D_{i}\;,D_{i+1}$ are not consecutive time instants in $D_{S}$ and this case has to be excluded from further reasoning. Now, assume that I(t)≠0 for all $t\in \left(D_{i},D_{i+1}\right)$ and $\dot{\alpha }\left(t\right)\neq 0$, otherwise, if $\dot{\alpha }\left(t\right)=0$ then $t\in D_{s}$ and $D_{i}\;,D_{i+1}$ are not consecutive time instants in $D_{S}$. Thus, $\alpha \left(t\right)=\alpha \left(D_{i}\right)+\int_{D_{i}}^{t}\dot{\alpha }\left(\tau \right)d\tau =\int_{D_{i}}^{t}\dot{\alpha }\left(\tau \right)d\tau$ for all $t\in \left(D_{i},D_{i+1}\right)$. Since $\dot{\alpha }\left(t\right)\neq 0$ for all $t\in \left(D_{i},D_{i+1}\right)$, it has no sign change in $\left(D_{i},D_{i+1}\right)$ so that $\underset{t\to D_{i+1}^{-}}{lim}\alpha \left(t\right)\neq 0$ and since $\alpha :R_{0+}\to R_{0+}$ is continuous then $\alpha \left(D_{i+1}\right)\neq 0$ which contradicts that $D_{i+1}\in D_{S}$. It has been proved that Case 1 is impossible $0\leq L_{i}<D_{i}<D_{i+1}<L_{i+1}$ cannot happen.

*Case 2*: Assume now that there are two consecutive zeros of $\ddot{I}\left(t\right)$ between two consecutive zeros of $\dot{I}\left(t\right)$, that is $0\leq D_{i}<L_{i}<L_{i+1}<D_{i+1}$ for some consecutive time instants $D_{i}\;,D_{i+1}$ in $D_{S}$ and some two consecutive time instants $L_{i}\;,L_{i+1}$ in $L_{S}$. Then, α(t)≠0 for all $t\in \left(L_{i},\;L_{i+1}\right)$ since, otherwise, there exists some $t\in \left(L_{i},\;L_{i+1}\right)$ such that $t\in D_{S}$, and then the previously claimed constraint $0\leq D_{i}<L_{i}<L_{i+1}<D_{i+1}$ does not hold, and also $\dot{\alpha }\left(t\right)\neq -\alpha^{2}\left(t\right)<0$ for all $t\in \left(L_{i},\;L_{i+1}\right)$ since, otherwise, there exists some $t\in \left(L_{i},\;L_{i+1}\right)$ such that $t\in L_{S}$ and then $L_{i}$ and $L_{i+1}$ are not two consecutive time instants in $L_{S}$ as claimed. Also, note that.

$\dot{\alpha }\left(L_{i}\right)+\alpha^{2}\left(L_{i}\right)=\dot{\alpha }\left(L_{i}\right)+\alpha^{2}\left(L_{i}\right)=0$ with $\alpha \left(L_{i}\right)\neq 0$ and $\alpha \left(L_{i+1}\right)\neq 0$ since $L_{i}\;,L_{i+1}\notin D_{S}$. But then, by continuity arguments on $\dot{\alpha }\left(t\right)+\alpha^{2}\left(t\right)$, there is a change of sign point $t\in \left(L_{i},\;L_{i+1}\right)$ which zeroes this function which contradicts $\dot{\alpha }\left(t\right)\neq -\alpha^{2}\left(t\right)<0$ for all $t\in \left(L_{i},\;L_{i+1}\right)$. Then, Case 2 is impossible so that $0\leq D_{i}<L_{i}<L_{i+1}<D_{i+1}$ cannot happen and Property (ii) has been proved. □

**Proof** **of** **Property** **(iii).** Assume that, contrarily to the statement, $D_{1}\leq L_{1}$. If $L_{1}=D_{1}$ then $\dot{I}\left(L_{1}\right)=\ddot{I}\left(L_{1}\right)$ and the equilibrium point is reached in finite time what is impossible, since $I_{0}>0$, for a non-trivial solution of a continuous-time first-order differential equation with continuous-time parameterization. Then, $L_{1}=D_{1}$ is impossible. Now, assume that $L_{1}>D_{1}$ and $0=\dot{I}\left(L_{1}\right)=\dot{I}\left(D_{1}\right)+\int_{L_{1}}^{D_{1}}\ddot{I}\left(\tau \right)d\tau =\int_{L_{1}}^{D_{1}}\ddot{I}\left(\tau \right)d\tau$ with $\ddot{I}\left(L_{1}\right)=0$ and then it exists some $L_{2}\in \left(L_{1},D_{1}\right)$ such that $\ddot{I}\left(L_{2}\right)=0$ and $L_{2}\in L_{S}$. As a result, there is $D_{1}>L_{2}>L_{1}$ and then there are two consecutive undulation time instants what contradicts Property (ii). As a result, $D_{1}>L_{1}$ as claimed. □

**Remark** **1.** *In Theorem 2, note that the sets* $D_{S}$ *and* $L_{S}$ *have the following properties:*

*They are nonempty so that there is at least one* $D\in D_{S}$ *such that* α(D)=0 *implying that* $\dot{I}\left(D\right)=0$ *and at least one* $L\in L_{S}$ *such that* $\dot{\alpha }\left(L\right)=-\alpha^{2}\left(L\right)$ *implying that* $\ddot{I}\left(L\right)=0$ *. Otherwise, the infection could converge asymptotically to zero as time goes to infinity but it would not have finite zeros,*

*They are sets of zero Lebesgue measure so that they are denumerable discrete sets of strictly ordered isolated real points, for any real numbers,*

*They fulfill that* $cardL_{S}=cardD_{S}+\vartheta$ *with* ϑ={0, 1} *so that they are of either identical finite or infinite cardinal or the cardinal of* $L_{S}$ *is finite and exceeds that of* $D_{S}$ *by one,*

*If* ϑ=0 *then* $card\left(D_{S}\right)=card\left(L_{S}\right)\leq {ℵ}_{0}$ *, that is, if both sets have infinity cardinal or identical finite one then any ordered points of* $L_{S}$ *and* $D_{S}$ *alternate.*

On the other hand, note that: 

Equation (4) establishes that $D_{S}$ is the set of zeros of α(t). At those zeros, the first-time derivative of the infection function is zeroed from (1) without such a function being necessarily zero while on the other hand, Equation (5) is a nonzero real constant for any finite undulation time instant $L_{i}\leq \bar{L}$ of $I:R_{0+}\to R_{0+}$ zeroing the second derivative of the infection function according to (2) which holds if c=K from (5). The fact that (5) is constant follows easily under periodicity conditions of the same or integer multiple/submultiple periods of g(t) and h(t).

Since $\alpha :R_{0+}\to R_{0+}$ has no finite zero coincident with a zero of its first time-derivative, by hypothesis, then $g\left(L_{i}\right)=E\Leftrightarrow h\left(L_{i}\right)\dot{g}\left(L_{i}\right)\neq 0$ since c≠0 from inspection of (8)–(9). This is equivalent to $D_{S}\cap L_{S}=\emptyset$, that is, the finite zeros which make zero $\dot{I}\left(t\right)$ and which do not make zero I(t) do not make zero either $\ddot{I}\left(t\right)$. However, $\ddot{I}\left(t\right)=0$ if $I\left(t\right)=\dot{I}\left(t\right)=0$ from (2), provided that $\alpha :R_{0+}\to R_{0+}$ is twice everywhere continuously differentiable in [0, +∞) but this can only happen as time tends to infinity for certain structures of g(t) and h(t). Note that the constraint (5) also implies that the auxiliary functions $g,\;h:R_{0+}\to R_{0+}$ used to define the function $\alpha :R_{0+}\to R_{0+}$ in (1) fulfill the constraint $h\left(L_{i}\right)\dot{g}\left(L_{i}\right)\neq ln\left(g\left(L_{i}\right)/E\right)\dot{h}\left(L_{i}\right)g\left(L_{i}\right)$; $\forall L_{i}\in L_{S}$.

By examining Definitions 1 and 3 and Lemma 1, it turns out that the set $L_{S}$ of undulation points of I(t) includes but, maybe non-properly, the set of its inflection points. However, it suffices to give some further weak conditions on $\alpha :R_{0+}\to R_{0+}$, that is, on $g,\;h:R_{0+}\to R_{0+}$ to guarantee that every undulation point of I(t) is also an inflection point. Some such conditions are discussed in the next corollary.

**Corollary** **2.** *The following properties hold:*

(**i**) *Assume that:*
  $$\underset{\varepsilon \to 0^{+}}{lim\;sup}\left[\theta \left(L_{i}+\varepsilon \right)\theta \left(L_{i}-\varepsilon \right)\right]<0;\;\forall L_{i}\in L_{S}$$
  *where:*
  $$\theta \left(t\right)=h\left(t\right)\dot{g}\left(t\right)-g\left(t\right)\dot{h}\left(t\right)ln\left(g\left(t\right)/E\right);\;\forall t\in R_{0+}$$
*Then, the set* $L_{S}$ *of undulation points of* I(t) *is the set of its inflection points.*
(**ii**) *Assume that* $f,g:R_{0+}\to R_{0+}$ *are twice continuously differentiable at each undulation point* $L_{i}\in L_{S}$ *. Then, the sets of undulation points and that of the inflection points of* I(t) *coincide if*
  $$\frac{h^{3}\left(L_{i}\right)\left(g\left(L_{i}\right)\;\ddot{g}\left(L_{i}\right)-{\dot{g}}^{2}\left(L_{i}\right)\right)}{h^{4}\left(L_{i}\right)g^{2}\left(L_{i}\right)}\neq \;\frac{2}{h^{3}\left(L_{i}\right)}\left({ln}^{3}\frac{g\left(L_{i}\right)}{E}+\dot{h}\left(L_{i}\right)ln\frac{g\left(L_{i}\right)}{E}\right);\;\forall L_{i}\in L_{S}$$

**Proof.**  Note that $\ddot{I}\left(t\right)=\left(\alpha^{2}\left(t\right)+\dot{\alpha }\left(t\right)\right)I\left(t\right)$, $\forall t\in R_{0+}$ so that $\ddot{I}\left(L_{i}\pm \varepsilon \right)=\left(\alpha^{2}\left(L_{i}\pm \varepsilon \right)+\dot{\alpha }\left(L_{i}\pm \varepsilon \right)\right)I\left(L_{i}\pm \varepsilon \right)$. Since $L_{i}>0$, g(t)h(t)>0 if t>0 and $\underset{\varepsilon \to 0}{lim}I\left(L_{i}\pm \varepsilon \right)=I\left(L_{i}\right)$, since I(t) is continuous, one gets that $\underset{\varepsilon \to 0^{+}}{lim\;sup}\left[\ddot{I}\left(L_{i}+\varepsilon \right)\ddot{I}\left(L_{i}-\varepsilon \right)\right]<0$ if and only if $\underset{\varepsilon \to 0^{+}}{lim\;sup}\left[\theta \left(L_{i}+\varepsilon \right)\theta \left(L_{i}-\varepsilon \right)\right]<0$. Property (i) has been proved.

On the other hand, if $f,g:R_{0+}\to R_{0+}$ are twice continuously differentiable at each undulation point $L_{i}\in L_{S}$ of I(t), then $\ddot{f},\ddot{g}$ exist in $L_{S}$. Then, defining $\hat{\alpha }\left(t\right)=-c^{-1}\alpha \left(t\right)=\frac{ln\left(g\left(t\right)/E\right)}{h\left(t\right)}$; $\forall t\in R_{0+}$ yields:

$$\dot{\hat{\alpha }}\left(t\right)=\frac{h\left(t\right)\dot{g}\left(t\right)-g\left(t\right)\dot{h}\left(t\right)ln\left(g\left(t\right)/E\right)}{h^{2}\left(t\right)g\left(t\right)};\;\forall t\in L_{S}$$

$$\ddot{\hat{\alpha }}\left(t\right)=\frac{h^{3}\left(t\right)\left(g\left(t\right)\ddot{g}\left(t\right)-{\dot{g}}^{2}\left(t\right)\right)/g^{2}\left(t\right)+\dot{h}\left(t\right)\dot{g}\left(t\right)/g\left(t\right)-2h\left(t\right)\dot{h}\left(t\right)ln\left(g\left(t\right)/E\right)}{h^{4}\left(t\right)};\;\forall t\in L_{S}$$

$$\ddot{I}\left(t\right)=\left(\alpha^{2}\left(t\right)+\dot{\alpha }\left(t\right)\right)I\left(t\right)\Rightarrow \dddot{I}\left(t\right)=0\;\mathrm{with}\;\alpha^{2}\left(t\right)=-\dot{\alpha }\left(t\right)\;\mathrm{and}\;I\left(t\right)>0;\;\forall t\in L_{S}$$

$$\dddot{I}\left(t\right)=\left(\alpha^{2}\left(t\right)+\dot{\alpha }\left(t\right)\right)\dot{I}\left(t\right)+\left(2\alpha \left(t\right)\dot{\alpha }\left(t\right)+\ddot{\alpha }\left(t\right)\right)I\left(t\right)\Rightarrow \dddot{I}\left(t\right)=\left(2\alpha \left(t\right)\dot{\alpha }\left(t\right)+\ddot{\alpha }\left(t\right)\right)I\left(t\right)=\left(\ddot{\alpha }\left(t\right)-2\alpha^{3}\left(t\right)\right)I\left(t\right);\;\forall t\in L_{S}$$

Since I(t)>0; $\forall t\in R_{0+}$ then $\dddot{I}\left(t\right)\neq 0$; $\forall t\in L_{S}$ if and only if $\ddot{\alpha }\left(t\right)\neq 2\alpha^{3}\left(t\right)$; $\forall t\in L_{S}$, equivalently, if and only if $\ddot{\hat{\alpha }}\left(t\right)\neq 2{\hat{\alpha }}^{3}\left(t\right)$; $\forall t\in L_{S}$ which is fully equivalent to the condition of Property (ii). The proof is complete.  □

**Remark** **2.** *Note that Theorem 2 applies, in particular, to the case when there are equilibrium points with the initial conditions being distinct from such points. It can be also extended by including the above case by redefining finite discrete sets of the zeros of* $\dot{I}\left(t\right)$ *and* $\ddot{I}\left(t\right)$ $D_{S}\to D_{S}\cap \left[0,\;L\right]$ *¸* $L_{S}\to L_{S}\cap \left[0,\;L\right]$ *for any given* L∈[0, ∞) *in the sense that the eventual zeros at finite time of* $\dot{I}\left(t\right)$ *and* $\ddot{I}\left(t\right)$ *alternate although an equilibrium points has not still been reached provided that it exists.*

Inspired in Theorem 2, some conditions are discussed in the next result which imply that the first undulation point of the infection evolution function (i.e., the first zero of its second-time derivative) precedes the first zero of its first time-derivative. It is not required that the infection has necessarily a disease-free equilibrium point or that it might be oscillatory leading to successive zeros of its time- derivative along time.

**Theorem** **3.** *Assume that the function* $\alpha \left(t\right)=-\frac{cln\left(g\left(t\right)/E\right)}{h\left(t\right)}$ *, where* $c\;,\;E\in R_{+}$ *and* $g,h:R_{0+}\to R_{0+}$ *are everywhere continuous and time-differentiable and satisfy the constraints:*

*(1)*  g(t)<E *;* ∀t∈[0, D) *,* g(D)=E
*(2)*  $\dot{g}\left(0\right)<\;\left(\frac{c{ln}^{2}\left(g\left(0\right)/E\right)}{h\left(0\right)}-\frac{\left|ln\left(g\left(0\right)/E\right)\right|}{h\left(0\right)\dot{h}\left(0\right)}\right)g\left(0\right)$
*(3)*  g(t)>0 *and* h(t)>0 *if* t>0
*(4)*  $\frac{ln\left(g\left(0\right)/E\right)}{h\left(0\right)}\neq 0$

*Assume also that* $I_{0}\;>0$ *. Then,* $min\left(I\left(t\right),\;\dot{I}\left(t\right)\right)>0$ *;* ∀t∈[0, D) *;* $\dot{I}\left(D\right)=0$ *and there is some* L∈(0, D) *such that* $\ddot{I}\left(t\right)\neq 0$ *;* ∀t∈[0, L) *and* $\ddot{I}\left(L\right)=0$ *.*

**Proof.**  Note from the definition of α(t), (1), (2) and the given constraints 1 and 2 that

$$\dot{\alpha }\left(0\right)=-\frac{c}{h\left(0\right)}\left(\frac{1}{h\left(0\right)\dot{h}\left(0\right)}\left|ln\frac{g\left(0\right)}{E}\right|+\frac{\dot{g}\left(0\right)}{g\left(0\right)}\right)$$

α(0)>0, since 0≤g(0)<E, α(D)=0, since g(D)=E), $\alpha^{2}\left(0\right)+\dot{\alpha }\left(0\right)>0$, from the condition 2 since α(0)>0 and since $\alpha :R_{0+}\to R_{0+}$ is continuous and time-differentiable since $g,h:R_{0+}\to R_{0+}$ are everywhere continuous and time-differentiable. Note also that, from the given assumptions and constraints, $min\left(I_{0},\;{\dot{I}}_{0},{\ddot{I}}_{0}\;\right)>0$ since $I_{0}>0$ by hypothesis, ${\dot{I}}_{0}=\alpha \left(0\right)\;I_{0}>0$ and ${\ddot{I}}_{0}=\left(\alpha^{2}\left(0\right)+\dot{\alpha }\left(0\right)\right)I_{0}>0$. Furthermore, $\dot{I}\left(D\right)=\alpha \left(D\right)I\left(D\right)=0$. From the constraint 3 and the continuity of $g,h:R_{0+}\to R_{0+}$, one has that $\alpha ,\dot{\alpha },\ddot{\alpha }:R_{0+}\to R_{0+}$ are continuous and bounded on (0,+∞), $\dot{I}\left(t\right)>0$; ∀t∈[0, D) and $\ddot{I}\left(t\right)>0$; $\forall t\in \left[0,\;L_{0}\right)$ and some $L_{0}\in R_{+}$. Furthermore since c>0 and $\frac{ln\left(g\left(0\right)/E\right)}{h\left(0\right)}\neq 0$, from the constraint 4, g(t)<E; ∀t∈[0, D), from the constraint 1, and g(t)>0 and h(t)>0 if t>0, from the constraint 3. Then α(t)>0; ∀t∈[0, D). Since $g,h:R_{0+}\to R_{0+}$ are continuous and positive on any bounded interval [0, T) then α(t) is positive and finite on [0, D). It is now proved that t=D is the first zero of $\dot{I}\left(t\right)$. Assume that this is not the case so that there is some $D_{1}<D$ such that $\dot{I}\left(D_{1}\right)=0$, with $\alpha \left(D_{1}\right)\neq 0$, and $\dot{I}\left(t\right)>0$; $\forall t\in \left[0,\;D_{1}\right)$. Then $I\left(D_{1}\right)=\dot{I}\left(D_{1}\right)=\dot{I}\left(D_{1}\right)=0$ from (2) and the infection extinguishes in a finite time $D_{1}<D$. This leads to a contradiction since $I\left(D_{1}\right)=I_{0}+\int_{0}^{D_{1}}\dot{I}\left(\tau \right)d\tau >0$ since $I_{0}>0$ and $\dot{I}\left(t\right)>0$; $\forall t\in \left[0,\;D_{1}\right)$. Therefore, if $D_{1}<D$ such that $\dot{I}\left(D_{1}\right)=0$ then $I\left(D_{1}\right)>0$. But then $\alpha \left(D_{1}\right)=\dot{I}\left(D_{1}\right)/I\left(D_{1}\right)=0$ from (1) which contradicts that α(t)≠0; ∀t∈[0, D). As a result, t=D is the first zero of $\dot{I}\left(t\right)$ and there is no $D_{1}<D$ such that $I\left(D_{1}\right)=0$. Since $I,\dot{I}:R_{0+}\cap \left[0,\;D\right]\to R_{0+}$ are continuous with $\dot{I}\left(t\right)>0$; ∀t∈[0, D) and $\dot{I}\left(D\right)=0$ and $\ddot{I}\left(t\right)$; $\forall t\in \left[0,\;L_{0}\right)$ and some $L_{0}\in R_{+}$ then there is some L∈(0, D) such that $\ddot{I}\left(L\right)=0$. Assume that this is not the case. Then, $0=\dot{I}\left(D\right)={\dot{I}}_{0}+\int_{0}^{D}\ddot{I}\left(\tau \right)d\tau >0$. Hence, a contradiction arises. Thus, there is some L∈(0, D) such that $\ddot{I}\left(L\right)=0$. □

**Remark** **3.** *Note that, under all the conditions of Theorem 3,* α(t)>0 *;* ∀t∈[0,D) *and* α(D)=0 *. Furthermore, the first zero of* $\dot{I}\left(t\right)=0$ *occurs at* t=D *, there is no* t<D *such that* $I\left(t\right)=\dot{I}\left(t\right)=0$ *and there is some* L<D *such that* $\ddot{I}\left(L\right)=0$ *.*

The following example describes the basic model proposed in [11] under a first-order differential equation for the infection evolution without any entropy considerations at this stage:

**Example** **1.** *The function* α(t)=−c ln(t/D)/t *, for some* D>0 *, proposed in [11] satisfies all the conditions of Theorem 3 with* h(t)=g(t)=t *and* E=D *. It satisfies, in addition, that* α(0)=+∞ *. This function satisfies also the given further conditions of Theorem 2* g(0)=h(0)=0 *with* $\underset{t\to 0}{lim}\frac{ln\left(g\left(t\right)/E\right)}{h\left(t\right)}\leq -\varepsilon$ *.*

*Note that the condition* α(0)>0 *of Theorem 3 avoids that* ${\dot{I}}_{0}=0$ *if* $I_{0}\neq 0$ *so that* t=0 *is a zero of* $\dot{I}\left(t\right)$ *.*

It can be argued that the proposed basic model (1) is a very simple time-varying differential equation of first-order which describes the infective population time-evolution. Note that the use of appropriate particular structures in the definition of the time-varying coefficient α(t) can take care of the eventual incorporation of the necessary supplementary environment information to make such an equation well-posed to practically describe a concrete disease evolution through time. The incorporation which can be incorporated is the eventual couplings of the infectious subpopulation with another ones (such as the susceptible, recovered or vaccinated subpopulations and their associated dynamics) or the information about the feedback information controls in more elaborated models. The next section develops some work in this direction.

## 3. Further Examples of Linking the Basic Model to Some Existing Epidemic Models Incorporating Other Subpopulations

The infection description via (1) assumes implicitly that it has a first-order dynamics. It has been argued that α(t) in (1) contains the information about the controls and other coupled subpopulations influencing the disease evolution through time. It can be of interest to discuss its application to infection descriptions described by differential equations of orders higher than one which is a very common situation in disease transmission mathematical models. 

It is now seen how a well-known epidemic model can be also discussed under the point of view of Theorem 3. In the subsequent example, the above characterization, based on the first zero of infection evolution time-derivative and on the undulation point of the infection evolution, is used for a model with three subpopulations via an appropriate choice of g(t) and h(t) in the definition of α(t).

**Example** **2.** *Consider the following SIR model without demography [30]:*

(10) $$\dot{S}\left(t\right)=-\beta S\left(t\right)I\left(t\right);\;\dot{I}\left(t\right)=\left(\beta S\left(t\right)-\gamma \right)I\left(t\right);\;\dot{R}\left(t\right)=\gamma I\left(t\right);\;\forall t\in R_{0+}$$

*where* S(t) *,* I(t) *and* R(t) *are, respectively, the susceptible, infectious and recovered (or immune) subpopulations, under nonzero initial conditions being subject to* min(S(0), I(0), R(0))≥0 *, where* β *is the coefficient transmission rate and* γ *is the removal or recovery rate (its inverse* $\gamma^{-1}$ *being the average infectious period). The mathematical study of this model and their variants is not easy as seen in [30,40]. First, note that the total population* $N\left(t\right)=S\left(t\right)+R\left(t\right)+I\left(t\right)=S_{0}+R_{0}+I_{0}$ *;* $\forall t\in R_{0+}$ *is constant for all time. The basic reproductive ratio (or reproduction number) is* $R_{{}_{*}}=\beta /\gamma$ *and, if* $S_{0}\leq R_{{}_{*}}^{-1}$ *, then* ${\dot{I}}_{0}\leq 0$ *while if* $S_{0}>R_{{}_{*}}^{-1}$ *, it becomes endemic for all time since* ${\dot{I}}_{0}>0$ *. The solution of (10) becomes in closed form:*

(11) $$S\left(t\right)=e^{-\beta \int_{0}^{t}I\left(\tau \right)d\tau }S_{0};\;I\left(t\right)=e^{\int_{0}^{t}\left(\beta S\left(\tau \right)-\gamma \right)d\tau }I_{0};\;R\left(t\right)=S_{0}+R_{0}+I_{0}-S\left(t\right)-I\left(t\right);\;\forall t\in R_{0+}$$

Note that by combining the above equations that:

(12) $$S\left(t\right)=e^{-\beta I_{0}\int_{0}^{t}e^{\int_{0}^{\tau }\left(\beta S\left(\sigma \right)-\gamma \right)d\sigma }d\tau }S_{0};\;I\left(t\right)=e^{\int_{0}^{t}\left(\beta e^{-\beta \int_{0}^{\tau }I\left(\sigma \right)d\sigma }S_{0}-\gamma \right)d\tau }I_{0}$$

Note from (11) that $S:R_{0+}\to R_{0+}$ is non-increasing so that there exists a susceptible equilibrium subpopulation $S_{e}=\underset{t\to \infty }{lim}S\left(t\right)\leq S_{0}$ for any given non-negative initial conditions. Note also from (10) that $\dot{N}\left(t\right)=0$ and then $N\left(t\right)=N_{0}$; $\forall t\in R_{0+}$ Note that If $I_{0}=0$ then I(t)=0, $S\left(t\right)=S_{0}$ and $R\left(t\right)=R_{0}=N_{0}-S_{0}$; $\forall t\in R_{0+}$. We examine three cases for $I_{0}>0$:

*Case (a)* if $S_{0}<R_{{}_{*}}^{-1}$ then $S\left(t\right)\leq S_{0}$ and βS(t)−γ<0; $\forall t\in R_{0+}$, then $I\left(t\right)\to 0,S\left(t\right)\to S_{e}$ and $R\left(t\right)\to R_{e}=N_{0}-S_{e}$ as t→∞. Since $S:R_{0+}\to R_{0+}$ is non-increasing, $S_{e}\leq S_{0}<R_{{}_{*}}^{-1}$. This implies that $\underset{t\to \infty }{lim}\int_{0}^{t}\left(\beta S\left(\tau \right)-\gamma \right)d\tau =-\infty$ and $\dot{I}\left(t\right)=-\lambda \left(t\right)I\left(t\right)\leq -\lambda_{a}I\left(t\right)$, I(t)→0 at exponential rate as t→∞ for some $\lambda_{a}>0$ from (10) and (11) since $I_{0}-I\left(t\right)\geq \lambda_{a}\int_{0}^{t}I\left(\tau \right)d\tau$ so that $\int_{0}^{\infty }I\left(\tau \right)d\tau \leq I_{0}/\lambda_{a}<+\infty$. Then, $I:R_{0+}\to R_{0+}$ is integrable on [0,∞). Thus, $C=\beta \int_{0}^{\infty }I\left(t\right)dt<+\infty$ so that $S_{e}=e^{-\beta \int_{0}^{\infty }I\left(t\right)dt}S_{0}=e^{-C}S_{0}>0$ (then there is a nonzero susceptible equilibrium level) and $R_{e}=N_{0}-S_{e}<N_{0}$.

*Case (b)* if $S_{0}=R_{{}_{*}}^{-1}$ then $S\left(t\right)\to S_{e}\leq S_{0}=\gamma /\beta$ as t→∞ since $S:R_{0+}\to R_{0+}$ is non-increasing and then it converges to $S_{e}$ satisfying $0\leq S_{e}\leq S_{0}$. By inspection of the second equation of (11), it also follows that $I\left(t\right)\to I_{e}$ and $R\left(t\right)\to R_{e}$ as t→∞ satisfying $I_{e}\geq 0$ and $R_{e}\geq 0$. Assume that $I_{e}>0$ then $S_{e}=0$ from the first equation of (11). But if $S_{e}=0$ then $I_{e}=0$ since then $I:R_{0+}\to R_{0+}$ is strictly decreasing on $\left[t_{a},\;\infty \right)$ for some finite $t_{a}>0$ from the second equation of (11). Hence, a contradiction to $I_{e}>0$ follows implying that $I_{e}=0$ if $S_{e}=0$. Now, assume that $\gamma /\beta >S_{e}>0$. Then, from the second equation of (11), $I\left(t\right)\to I_{e}=0$ as t→∞. But then $S_{e}>0$, from the first equation of (12), since $\gamma /\beta >S_{e}$ if $I_{0}>0$ and then $R_{e}=N_{0}-S_{e}$. From the second equation of (12) and, under a similar reasoning as that of Case a, $I:R_{0+}\to R_{0+}$ is integrable on [0,∞) and $S_{e}>0$. In summary, if $S_{0}=R_{{}_{*}}^{-1}=\gamma /\beta$ and $I_{0}>0$ then I(t)→0,S(t)→0 and $R\left(t\right)\to N_{0}=S_{0}+R_{0}+I_{0}$ as t→∞ in the same way as in Case a if $S_{0}\leq R_{{}_{*}}^{-1}$.

Case (c) if $S_{0}>R_{{}_{*}}^{-1}$ then ${\dot{I}}_{0}>0$ from (10) and $S:R_{0+}\to R_{0+}$ is increasing on some interval $\left[0,t_{0}\right]$. The fact that $I:R_{0+}\to R_{0+}$ is strictly increasing on some initial time interval is of interest from the point of view of hospital management of availability of beds and other sanitary specific means in the event that the disease might have a relevant number of seriously infected individuals. Since $S:R_{0+}\to R_{0+}$ is non-increasing then either $I\left(t\right)\to I_{e}=S_{0}+I_{0}+R_{0}=N_{0}$, $S\left(t\right)\to S_{e}=0$ and $R\left(t\right)\to R_{e}=0$ as t→∞ or $S\left(t\right)\to S_{e}\in {\left(0,\;R_{{}_{*}}^{-1}\;\right]}^{}$ as t→∞ from (11) since $S:R_{0+}\to R_{0+}$ is non-increasing. The firs possibility $I\left(t\right)\to I_{e}=N_{0}$ is unfeasible since from the first equation of (11) I(t)→∞ as t→∞. Then, $S\left(t\right)\to S_{e}\in {\left(0,\;R_{{}_{*}}^{-1}\;\right]}^{}$ as t→∞. Now, first, assume that $S_{e}\in \left(\gamma /\beta ,\;R_{{}_{*}}^{-1}\right]$. Then, from the first equation of (12), S(t)→0 as t→∞. Then, $S_{e}=0$ which contradicts that $S_{e}>\gamma /\beta \;$, As a result, $0\leq S_{e}\leq \gamma /\beta \;$. Now, assume that $S_{e}=0$. Then, from (11), $I\left(t\right)\to I_{e}=0$ and $I:R_{0+}\to R_{0+}$ being square-integrable, and following a similar argument as that of Cases a–b, one again concludes that $S_{e}>0$ so that $S_{e}\in \left(0,\;\gamma /\beta \;\right]$ and $R_{e}=N_{0}-S_{e}$, as a result. But, since $S_{e}\leq \gamma /\beta$ then $I_{e}=0$ from (11) since $I:R_{0+}\to R_{0+}$ is strictly decreasing after some finite time instant $t_{0}$ and integrable on [0,∞) and a following again the reasoning of Cases a–b, one concludes that $S_{e}>0$. As a result, if $S_{0}>R_{{}_{*}}^{-1}$ and $I_{0}>0$, then $I_{e}=0$, $S_{e}>0$ and $R_{e}=N_{0}-S_{e}$. Thus, the relevant conclusions on the disease- free equilibrium point which is a disease- free one are similar for the three above cases.

On the other hand, since $S:R_{0+}\to R_{0+}$ it exists a finite t=D>0 such that $S\left(D\right)=R_{{}_{*}}^{-1}=\gamma /\beta$ and $\dot{I}\left(D\right)=\alpha \left(D\right)I\left(D\right)=\left(\beta S\left(D\right)-\gamma \right)I\left(D\right)=0$, $I\left(D\right)=e^{\int_{0}^{D}\left(\beta S\left(\tau \right)-\gamma \right)d\tau }I_{0}\neq 0$, if $I_{0}\neq 0$ and, furthermore,

(13) $$\begin{array}{c} \ddot{I}\left(D\right)=\left(\beta \dot{S}\left(D\right)-\gamma \right)I\left(D\right)+\left(\beta S\left(D\right)-\gamma \right)\dot{I}\left(D\right)\\ =\left(\beta \dot{S}\left(D\right)-\gamma \right)I\left(D\right)=-\beta^{2}S\left(D\right)I^{2}\left(D\right)-\gamma I\left(D\right)\\ =-\gamma \left(\beta \;e^{\int_{0}^{D}\left(\beta S\left(\tau \right)-\gamma \right)d\tau }I_{0}+1\right)e^{\int_{0}^{D}\left(\beta S\left(\tau \right)-\gamma \right)d\tau }I_{0}<0 \end{array}$$

and also:

(14) $${\ddot{I}}_{0}=-\beta^{2}S_{0}I_{0}^{2}+\left(\beta S_{0}-\gamma \right){\dot{I}}_{0}=I_{0}{\left[{\left(\beta S_{0}-\gamma \right)}^{2}-\beta^{2}S_{0}I_{0}\right]}^{}$$

and ${\ddot{I}}_{0}>0$ under the reasonable assumption that $I_{0}$ is sufficiently small (the initial numbers of infectious is usually very small in practice) satisfying $I_{0}<\frac{{\left(\beta S_{0}-\gamma \right)}^{2}}{\beta^{2}S_{0}}$. As a result, there is some time instant L∈(0, D) such that $\ddot{I}\left(L\right)=0$ so that it is an undulation point of $I:R_{0+}\to R_{0+}$. As a result, we find that if the basic reproduction number exceeds unity then the infection curve corresponding to the endemic solution has a minimum at a larger time instant that the one defining its undulation point. That situation corresponds to the situation of small initial infection force with reproduction number greater than one. On the other hand, if ${\ddot{I}}_{0}\leq 0$, then ${\dot{I}}_{0}>0$ does not hold.

Comparing the infectious subpopulation evolution to (1) and the structure of the function in Theorem 3 yields:

(15) $$\alpha \left(t\right)=\beta \;S\left(t\right)-\gamma =-\frac{cln\left(g\left(t\right)/E\right)}{h\left(t\right)}$$

(16) $$\dot{\alpha }\left(t\right)=\beta \;\dot{S}\left(t\right)=-c\frac{d}{dt}\left(\frac{ln\left(g\left(t\right)/E\right)}{h\left(t\right)}\right)$$

(17) $$=-\beta^{2}S\left(t\right)I\left(t\right)=-\frac{c}{h\left(t\right)}\left(\frac{1}{h\left(t\right)\dot{h}\left(t\right)}\left|ln\frac{g\left(t\right)}{E}\right|+\frac{\dot{g}\left(t\right)}{g\left(t\right)}\right);$$

$\forall t\in R_{0+}$. If one defines g(t)=t; $\forall t\in R_{0+}$ and $h\left(t\right)=\frac{cln\left(t/E\right)}{\gamma -\beta \;S\left(t\right)}$; $\forall t\in R_{0+}$, then $h\left(t\right)=\frac{c\left|ln\left(t/E\right)\right|}{\beta \;S\left(t\right)-\gamma }$; $\forall t\in R_{0+}$. It is easy to verify that these functions satisfy the conditions of Theorem 3.

In the case when the reproduction number is less than unity and it is an upper-bound of the normalized susceptible population, each primary infection generates, in average, less than one secondary one so that the infection extinguishes asymptotically. According to this particular model, also the susceptible subpopulation extinguishes asymptotically. See Case a referred to (11). Thus, the disease-free equilibrium point is ${\left(S_{df}^{*}\;,\;I_{df}^{*}\;,\;R_{df}^{*}\right)}^{T}={\left(0\;,\;0\;,\;N\right)}^{T}$. In this case, $I\left(t\right),\dot{I}\left(t\right),\ddot{I}\left(t\right)\to 0$ as t→∞ but there are no finite time instants of minimum and undulation of the infectious curve to the light of Theorem 3.

However, we can have a practical visualization of the disease removal by defining a design quadruple $\left(k_{1},k_{2},k_{3},\varepsilon \right)\in R_{+}^{4}$ and the following cut associate time instants:

(18) $$t_{Ii}\left(k_{i},\;\varepsilon \right)=min\left(\tau \in R_{0+}:\;\left|\frac{dI^{\left(i-1\right)}}{dt}\right|\leq k_{i}\varepsilon :\;t\in \left[\tau \;,\;+\infty \right)\right);\;i=1,2,3$$

Note that $t_{I2}\left(k_{2},\;\varepsilon \right)$ and $t_{I3}\left(k_{3},\;\varepsilon \right)$ generalize the roles of the time instants D and L, that is, the finite minimum infection and undulation time instants, respectively, within prescribed margins when those time instants do not exist.

**Example** **3.** *Consider Case a of Example 2 so that*$S\left(t\right)\leq S_{0}<\gamma /\beta$*leading to*$I\left(t\right)\to 0,S\left(t\right)\to S_{e}>0$*and*$R\left(t\right)\to R_{e}=N_{0}-S_{e}$*as*t→∞*and*I(t)>0*,*$\dot{I}\left(t\right)<0$*and*$\ddot{I}\left(t\right)<0$*are strictly decreasing on*[0, +∞)*. Take prescribed constants*ε∈(0,1)$k_{i}\geq 1$*for*i=1,2,3*. The solution trajectory converges to the disease-free equilibrium point at exponential rate. Then, one gets by combining (10)–(12) and (18) that:*

(19) $$\left|\int_{0}^{t_{I1}}\left(\gamma -\beta S\left(\tau \right)\right)d\tau \right|=\left|\int_{0}^{t_{I1}}\left(\gamma -\beta e^{-\beta \int_{0}^{\sigma }I\left(\sigma \right)d\sigma }S_{0}\right)d\tau \right|\leq lnI_{0}-lnk_{1}+\left|ln\varepsilon \right|;\;\forall t\in R_{0+}$$

(20) $$\left(\gamma -\beta e^{-\beta \int_{0}^{t_{I2}}I\left(\tau \right)d\tau }S_{0}\right)e^{-\int_{0}^{t_{I2}}\left(\gamma -\beta e^{-\beta \int_{0}^{\tau }I\left(\sigma \right)d\sigma }S_{0}\right)d\tau }I_{0}\leq k_{2}\varepsilon ;\;\forall t\in R_{0+}$$

(21) $${\left[\beta^{2}\;S\left(t\right)I\left(t\right)-{\left(\beta S\left(t\right)-\gamma \right)}^{2}\right]}^{}I\left(t\right)\leq k_{3}\varepsilon ¸\;\forall t\in R_{0+}$$

*implying that:*

(22) $$t_{I1}=min\left(t\in R_{0+}\;:\gamma t-\beta S_{0}\int_{0}^{t}e^{-\beta \int_{0}^{\sigma }I\left(\sigma \right)d\sigma }d\tau =lnI_{0}-lnk_{1}+\left|ln\varepsilon \right|\right)\geq \frac{1}{\gamma }\left(lnI_{0}-lnk_{1}+\left|ln\varepsilon \right|\right)$$

(23) $$\begin{array}{c} 2min\left(k_{2}\varepsilon I_{0},\;\beta S_{0}\right)e^{-\beta \int_{0}^{t_{I2}}I\left(\tau \right)d\tau }\\ \leq \eta \left(t_{I2}\right)=k_{2}\varepsilon e^{\int_{0}^{t_{I2}}\left(\gamma -\beta S\left(\tau \right)\right)d\tau }I_{0}+\beta e^{-\beta \int_{0}^{t_{I2}}I\left(\tau \right)d\tau }S_{0}\\ \leq \left(k_{2}\varepsilon I_{0}+\beta S_{0}\right)e^{\int_{0}^{t_{I2}}\left(\gamma -\beta S\left(\tau \right)\right)d\tau }I_{0} \end{array}$$

*which leads to:*

(24) $$\begin{array}{c} e^{\int_{0}^{t_{I2}}\left(\gamma -\beta S\left(\tau \right)+\beta I\left(\tau \right)\right)d\tau }\geq \frac{2min\left(k_{2}\varepsilon I_{0},\;\beta S_{0}\right)}{\left(k_{2}\varepsilon I_{0}+\beta S_{0}\right)I_{0}}\\ \Rightarrow t_{I2}\geq max\left(t>0:\int_{0}^{t}\left(\gamma -\beta S\left(\tau \right)+\beta I\left(\tau \right)\right)d\tau \right)=ln\left[\frac{2min\left(k_{2}\varepsilon I_{0},\;\beta S_{0}\right)}{\left(k_{2}\varepsilon I_{0}+\beta S_{0}\right)I_{0}}\right] \end{array}$$

(25) $$\begin{array}{c} e^{\int_{0}^{t_{I2}}\left(\beta S\left(\tau \right)-\beta I\left(\tau \right)-\gamma \right)d\tau }\leq \frac{\left(k_{2}\varepsilon I_{0}+\beta S_{0}\right)I_{0}}{2min\left(k_{2}\varepsilon I_{0},\;\beta S_{0}\right)}\\ \Rightarrow t_{I2}\leq min\left(t>0:\int_{0}^{t}\left(\beta S\left(\tau \right)-\beta I\left(\tau \right)-\gamma \right)d\tau \right)=ln\left[\frac{\left(k_{2}\varepsilon I_{0}+\beta S_{0}\right)I_{0}}{2min\left(k_{2}\varepsilon I_{0},\;\beta S_{0}\right)}\right] \end{array}$$

and:

$$-k_{3}\varepsilon \leq \ddot{I}\left(t\right)=\left(\beta S\left(t\right)-\gamma \right)\dot{I}\left(t\right)+\beta \dot{S}\left(t\right)I\left(t\right)={\left[{\left(\gamma -\beta S\left(t\right)\right)}^{2}-\beta^{2}S\left(t\right)I\left(t\right)\right]}^{}I\left(t\right)\leq k_{3}\varepsilon$$

*what implies that* $\left|\ddot{I}\left(t\right)\right|\leq k_{3}\varepsilon$*;* $\forall t\in \left[t_{I3},\;\infty \right)$ *such that:*

$$t_{I3}\geq max\left(t>0:{\left[{\left(\gamma -\beta S\left(t\right)\right)}^{2}-\beta^{2}S\left(t\right)I\left(t\right)\right]}^{}I\left(t\right)\right)\geq -k_{3}\varepsilon ,$$

$$t_{I3}\leq min\left(t>0:{\left[{\left(\gamma -\beta S\left(t\right)\right)}^{2}-\beta^{2}S\left(t\right)I\left(t\right)\right]}^{}I\left(t\right)\right)\leq k_{3}\varepsilon$$

**Example** **4.** *Consider the following SIS model with vaccination and antiviral or antibiotic controls:*

(26) $$\dot{S}\left(t\right)=\gamma I\left(t\right)-\beta S\left(t\right)I\left(t\right)-k_{V}S\left(t\right);\;\dot{I}\left(t\right)=\left(\beta S\left(t\right)-\gamma -k_{T}\right)I\left(t\right);\;\forall t\in R_{0+}$$

*subject to* $S\left(0\right)=S_{0}$ *,* $I\left(0\right)=I_{0}$ *with* $min\left(S_{0},\;I_{0}\;\right)\geq 0$ *where the vaccination and treatment feedback controls on the susceptible and infectious are, respectively,* $V\left(t\right)=k_{V}S\left(t\right)$ *and* $T\left(t\right)=k_{T}I\left(t\right)$ *with* $min\left(k_{V},\;k_{T}\right)\geq 0$ *. If it is assumed that the total population* $N\left(t\right)=N_{0}=S_{0}+I_{0}$ *;* $\forall t\in R_{0+}$ *is constant through time then there is a complementary recovered (or immune) subpopulation present which obeys the differential equation* $\dot{R}\left(t\right)=k_{V}S\left(t\right)+k_{T}I\left(t\right)$ *with* $R\left(0\right)=R_{0}=0$ *. The solution is:*

(27) $$\begin{array}{c} S\left(t\right)=e^{-\int_{0}^{t}\left(\beta I\left(\tau \right)+k_{V}\right)d\tau }S_{0}+\gamma \int_{0}^{t}e^{-\int_{\tau }^{t}\left(\beta I\left(\sigma \right)+k_{V}\right)d\sigma }I\left(\tau \right)d\tau \\ =e^{-k_{V}t}S_{0}-\int_{0}^{t}e^{-k_{V}\left(t-\tau \right)}\left(\beta S\left(\tau \right)-\gamma \right)I\left(\tau \right)d\tau \end{array}$$

(28) $$I\left(t\right)=e^{\beta \int_{0}^{t}S\left(\tau \right)d\tau }e^{-\left(\gamma +\;k_{T}\right)t}I_{0}$$

(29) $$R\left(t\right)=\int_{0}^{t}\left(k_{V}S\left(\tau \right)+k_{T}I\left(\tau \right)\right)d\tau$$

The following result links the above SIS model with a complementary recovered subpopulation to the generic one (1) under a minimum number of initial susceptible and sufficiently large number of initial infectious with initial growing rate.

**Theorem** **4.** *Assume that* $S_{0}>\;\frac{\gamma +k_{T}}{\beta }$ *,* $I_{0}<1+\frac{1}{\gamma }\left(k_{T}+k_{V}S_{0}\right)$ *and* ${\dot{I}}_{0}>\frac{\beta \left|{\dot{S}}_{0}\right|I_{0}}{\beta S_{0}-\gamma -k_{T}}$ *.*

*Then, the following properties hold:*

(**i**) ${\dot{S}}_{0}<0$ *and* ${\ddot{I}}_{0}>0$ *,*
(**ii**) S(t) *is strictly decreasing on* $\left[0,\;t_{Smin}\right]$ *with* $t_{Smin}=min\left(t\in R_{0+}\;:\;S\left(t\right)=\gamma /\beta \right)$ *,*
(**iii**) I(t) *is strictly increasing on* $\left[0,\;t_{Imax}\right]$ *, and*
$I_{max}=I\left(t_{max}\right)=max\left(I\left(t\right)\;:\;\;t\in \left[0\;,\;t_{Imax}\right],\;t_{I}{}_{max}=min\left(t\in R_{0+}:S\left(t\right)=\left(\gamma +k_{T}\right)/\beta \right)\right)$ *with* $t_{Imax}\geq t_{Smin}$ *,*
(**iv**) *There is* $t_{und}<t_{Imax}$ *which is an undulation and, furthermore, strict inflection time instant of* I(t) *,*
(**v**) *Assume, in addition, that*$I_{0}$*is large enough to satisfy*$I_{0}>\frac{\left(\gamma +k_{T}\right)k_{V}}{\left(\gamma -\beta \left(\gamma +k_{T}\right)\right)}e^{-\beta \int_{0}^{t_{Imax}}S\left(\tau \right)d\tau }e^{\left(\gamma +\;k_{T}\right)t_{Imax}}$*. Then, the epidemic model (26) can be written in the form (1) on*$\left[0,\;t_{Imax}\right]$*with the following function*$\alpha :\left[0,\;t_{Imax}\right]\to R_{0+}$:
  (30) $$\alpha \left(t\right)=\beta \left(e^{-\int_{0}^{t}\left(\beta I\left(\tau \right)+k_{V}\right)d\tau }S_{0}+\gamma \int_{0}^{t}e^{-\int_{\tau }^{t}\left(\beta I\left(\sigma \right)+k_{V}\right)d\sigma }I\left(\tau \right)d\tau \right)-\gamma -k_{T};\;t\in \left[0,\;t_{Imax}\right]$$
  *which is of the form*$\alpha \left(t\right)=-\frac{cln\left(g\left(t\right)/E\right)}{h\left(t\right)}$*with*$g:\left[0,\;t_{Imax}\right]\to \left[0,\;E\right]$*;*$\forall t\in \left[0,\;t_{Imax}\right]$*and any given*$E\in R_{+}$*and*$h\left(t\right)=\frac{c\left|ln\left(g\left(t\right)/E\right)\right|}{\beta \left(e^{-\int_{0}^{t}\left(\beta I\left(\tau \right)+k_{V}\right)d\tau }S_{0}+\gamma \int_{0}^{t}e^{-\int_{\tau }^{t}\left(\beta I\left(\sigma \right)+k_{V}\right)d\sigma }I\left(\tau \right)d\tau \right)-\gamma -k_{T}}$*;*$\forall t\in \left[0,\;t_{Imax}\right]$*.*
(**vi**) *The equilibrium points are* $S_{1}^{*}=I_{1}^{*}=0$ *,* $R_{1}^{*}=N_{0}$ *if* $k_{V}\neq 0$ *and* $k_{T}\geq 0$ *, and* $S_{2}^{*}=\frac{\gamma +k_{T}}{\beta }$ *,* $I_{2}^{*}=0$ *and* $R_{2}^{*}=N_{0}-\frac{\gamma +k_{T}}{\beta }$ *which is only reachable if* $k_{V}=0$ *since, otherwise,* $I_{2}^{*}=-\frac{k_{V}}{k_{T}}\frac{\gamma +k_{T}}{\beta }<0$ *.*

**Proof.**  Since $S_{0}>\;\frac{\gamma +k_{T}}{\beta }$ and $I_{0}<1+\frac{1}{\gamma }\left(k_{T}+k_{V}S_{0}\right)$ then ${\dot{I}}_{0}>0$ and ${\dot{S}}_{0}<0$. Also, ${\ddot{I}}_{0}=\beta {\dot{S}}_{0}I_{0}+\left(\beta S_{0}-\gamma -k_{T}\right){\dot{I}}_{0}=\left(\beta S_{0}-\gamma -k_{T}\right){\dot{I}}_{0}-\beta \left|{\dot{S}}_{0}\right|I_{0}>0$ if ${\dot{I}}_{0}>\frac{\beta \left|{\dot{S}}_{0}\right|I_{0}}{\beta S_{0}-\gamma -k_{T}}$. Property (i) has been proved. Furthermore, $S_{0}>\;\frac{\gamma +k_{T}}{\beta }\geq \frac{\gamma }{\beta }$ implies from (27) that S(t) is strictly decreasing on [0, t′] where $t\prime =min\left(t\in R_{0+}\;:\;S\left(t\right)=\gamma /\beta \right)$ what proves Property (ii) with $t_{Smin}=t\prime$. On the other hand and since $S:R_{0+}\to R_{0+}$ is continuous, there exists some t″∈[0, t′] such that $S\left(t\prime \prime \right)=\;\frac{\gamma +k_{T}}{\beta }$ with t″=t′ if and only if $k_{T}=0$. From (26), $\dot{I}\left(t^{\prime \prime }\right)=0$ and $\dot{I}\left(t\right)>0$ for t∈[0,t″) since ${\dot{I}}_{0}>0$. On the other hand, one has from (26) and (28) that:

$$\ddot{I}\left(t\prime \prime \right)=\left(\beta S\left(t\prime \prime \right)-\gamma -k_{T}\right)\dot{I}\left(t\prime \prime \right)+\beta \dot{S}\left(t\prime \prime \right)I\left(t\prime \prime \right)$$

$$=\beta \left[\beta \left(\gamma -\beta S\left(t\prime \prime \right)\right)I\left(t\prime \prime \right)-k_{V}S\left(t\prime \prime \right)\right]I\left(t\prime \prime \right)$$

$$=-{\left[\beta^{2}k_{T}I\left(t\prime \prime \right)+k_{V}\left(\gamma +k_{T}\right)\right]}^{}I\left(t\prime \prime \right)$$

$$=-{\left[\beta^{2}k_{T}e^{\beta \int_{0}^{t}S\left(\tau \right)d\tau }e^{-\left(\gamma +\;k_{T}\right)t}I_{0}+k_{V}\left(\gamma +k_{T}\right)\right]}^{}e^{\beta \int_{0}^{t\prime \prime }S\left(\tau \right)d\tau }e^{-\left(\gamma +\;k_{T}\right)t\prime \prime }I_{0}<0$$

and I(t) has a relative maximum $I_{max}$ at $t=t\prime \prime =t_{Imax}$ which is also the absolute maximum on $\left[0,t_{max}\right]$. Property (iii) has been proved. Note also that since $\ddot{I}\left(t\right)$ is continuous and ${\ddot{I}}_{0}>0$, there exists some $t_{und}<t\prime \prime$ such that $t_{und}$ is an undulation point of I(t). Note furthermore that

$$\ddot{I}\left(t_{und}\right)=\left(\beta S\left(t_{und}\right)-\gamma -k_{T}\right)\dot{I}\left(t_{und}\right)+\beta \dot{S}\left(t_{und}\right)I\left(t_{und}\right)=0$$

From Lemma 1(i), $\ddot{I}\left(t_{und}-\varepsilon \right)\ddot{I}\left(t_{und}+\varepsilon \right)<0$; ∀ε∈B(0,r) and some $r\in R_{+}$ implies that $t_{und}$ is also an inflection time instant of I(t). The equivalent logic contrapositive proposition establishes that:

$$\left[\forall r\in R_{+},\;\exists \;\varepsilon \in \left[0,\;r\right]:\ddot{I}\left(t_{und}-\varepsilon \right)\ddot{I}\left(t_{und}+\varepsilon \right)\geq 0\right]\Rightarrow \;[t_{und}\;\mathrm{is}\;\mathrm{not}\;\mathrm{an}\;\mathrm{inflection}\;\mathrm{time}\;\mathrm{instant}\;\mathrm{of}\;I\left(t\right)]$$

Then, if $\ddot{I}\left(t_{und}-\varepsilon \right)\ddot{I}\left(t_{und}+\varepsilon \right)<0$; ∀ε∈B(0,r) and some $r\in R_{+}$ then $t_{und}$ is in fact an inflection time instant of I(t). Assume that there is some arbitrarily small $\varepsilon \in R_{+}$ such that $\ddot{I}\left(t_{und}-\varepsilon \right)\ddot{I}\left(t_{und}+\varepsilon \right)\geq 0$

Then:

$\dot{I}\left(t_{und}+\varepsilon \right)=\ddot{I}\left(t_{und}\right)+\int_{0}^{\varepsilon }\ddot{I}\left(t_{und}+\tau \right)d\tau$; $\dot{I}\left(t_{und}-\varepsilon \right)=\ddot{I}\left(t_{und}\right)+\int_{0}^{-\varepsilon }\ddot{I}\left(t_{und}+\tau \right)d\tau$.

Since $\ddot{I}\left(t\right)$ is continuous on $\left[t_{und}-\varepsilon ,\;t_{und}+\varepsilon \right]$ and one gets that

$$\dot{I}\left(t_{und}+\varepsilon \right)-\dot{I}\left(t_{und}-\varepsilon \right)=\int_{0}^{\varepsilon }\ddot{I}\left(t_{und}+\tau \right)d\tau -\int_{0}^{-\varepsilon }\ddot{I}\left(t_{und}+\tau \right)d\tau$$

It is known that $0<\varepsilon_{I}\leq \dot{I}\left(t_{und}\right)<{\dot{I}}_{0}$ so that, for some arbitrarily small $\varepsilon \in R_{+}$ such that $\ddot{I}\left(t_{und}-\varepsilon \right)\ddot{I}\left(t_{und}+\varepsilon \right)\geq 0$, there are $\varepsilon_{1}\in \left[0,\;\varepsilon \right]$ and $\varepsilon_{2}\in R_{+}$ with $-\varepsilon_{2}\in \left[-\varepsilon ,\;0\right]$ such that the following joint constraints hold:

(1) $\dot{I}\left(t_{und}+\tau \right)>0$; $\forall \tau \in \left[-\varepsilon_{2},\;\varepsilon_{1}\right]\subset \left[-\varepsilon ,\;\varepsilon \right]$ with $\dot{I}\left(t\right)$ being strictly increasing on $\left[-\varepsilon_{2},\;\varepsilon_{1}\right]$.
(2) $\int_{0}^{\varepsilon_{1}}\ddot{I}\left(t_{und}+\tau \right)d\tau =\int_{0}^{-\varepsilon_{2}}\ddot{I}\left(t_{und}+\tau \right)d\tau$

Then, one gets from Condition 2 that:

$$\dot{I}\left(t_{und}+\varepsilon_{1}\right)-\dot{I}\left(t_{und}-\varepsilon_{2}\right)=\int_{0}^{\varepsilon_{1}}\ddot{I}\left(t_{und}+\tau \right)d\tau -\int_{0}^{-\varepsilon_{2}}\ddot{I}\left(t_{und}+\tau \right)d\tau =0$$

so that $\dot{I}\left(t\right)$ is not strictly increasing on $\left[-\varepsilon_{2},\;\varepsilon_{1}\right]$, hence a contradiction. As a result, the undulation time instant $t_{und}$ of I(t) is also a strict inflection time instant of I(t) since $\dot{I}\left(t_{und}\right)\neq 0$ since Lemma 1 (ii) holds and the first zero of $\dot{I}\left(t\right)$ occurs at $t_{Imax}>t_{und}$. Property (iv) has been proved. To prove Property (v), note that Equation (30) follows from (26)–(27). Now, we equalize (30) to (1) to get admissible functions $g,h:R_{0+}\to R_{0+}$ leading to:

(31) $$\alpha \left(t\right)=\beta \left(e^{-\int_{0}^{t}\left(\beta I\left(\tau \right)+k_{V}\right)d\tau }S_{0}+\gamma \int_{0}^{t}e^{-\int_{\tau }^{t}\left(\beta I\left(\sigma \right)+k_{V}\right)d\sigma }I\left(\tau \right)d\tau \right)-\gamma -k_{T}=-\frac{cln\left(g\left(t\right)/E\right)}{h\left(t\right)}$$

and note that $\alpha \left(0\right)=\beta S_{0}-\gamma -k_{T}>0$. Note also that $\alpha \left(0\right)=\frac{+\infty }{h\left(0\right)}$ from the use of (31) in (30) implies that h(0)=0 irrespective of g(t) while g(t) is chosen arbitrary and continuous time-differentiable subject to g(0)=0 and $\alpha \left(t_{Imax}\right)=0$, $g\left(t_{Imax}\right)=E$ (so that $ln\left(g\left(t_{Imax}\right)/E\right)=0$) with $h\left(t\right)=\frac{c/E}{\;\beta \gamma I\left(t\right)-\beta \left(\beta I\left(t\right)+k_{V}\right)S\left(t\right)}$ for $t\in \left[0,\;t_{Imax}\right]$.

Now, note that $h\left(t_{Imax}\right)$ is a primary (0/0)—type indetermination which is resolved through L´H $\hat{o}$ pital rule leading to:

$$\begin{array}{c} h\left(t_{Imax}\right)=\frac{c/g\left(t_{Imax}\right)}{\beta \dot{S}\left(t_{Imax}\right)}=\frac{c/E}{\;\beta \gamma I\left(t_{Imax}\right)-\beta \left(\beta I\left(t_{Imax}\right)+k_{V}\right)S\left(t_{Imax}\right)}\\ =\frac{c/\left(\beta E\right)}{\;\gamma I\left(t_{Imax}\right)-\left(\beta I\left(t_{Imax}\right)+k_{V}\right)\left(\gamma +k_{T}\right)} \end{array}$$

Since $I\left(t_{Imax}\right)=e^{\beta \int_{0}^{t_{Imax}}S\left(\tau \right)d\tau }e^{-\left(\gamma +\;k_{T}\right)t}I_{0}$ then for sufficiently large $I_{0}$ such that

$$I_{0}>\frac{\left(\gamma +k_{T}\right)k_{V}}{\left(\gamma -\beta \left(\gamma +k_{T}\right)\right)}e^{-\beta \int_{0}^{t_{Imax}}S\left(\tau \right)d\tau }e^{\left(\gamma +\;k_{T}\right)t_{Imax}}$$

then:

$$h\left(t\right)=\frac{c\left|ln\left(g\left(t\right)/E\right)\right|}{\beta \left(e^{-\int_{0}^{t}\left(\beta I\left(\tau \right)+k_{V}\right)d\tau }S_{0}+\gamma \int_{0}^{t}e^{-\int_{\tau }^{t}\left(\beta I\left(\sigma \right)+k_{V}\right)d\sigma }I\left(\tau \right)d\tau \right)-\gamma -k_{T}}$$

$$=\frac{cln\left(g\left(t\right)/E\right)}{\gamma +k_{T}-\beta \left(e^{-k_{V}t}S_{0}-\int_{0}^{t}e^{-k_{V}\left(t-\tau \right)}\left(\beta S\left(\tau \right)-\gamma \right)I\left(\tau \right)d\tau \right)}$$

fulfilling, in particular:

$$h\left(t_{Imax}\right)=\frac{c/\left(\beta E\right)}{\;\left(\gamma -\beta \left(\gamma +k_{T}\right)\right)It_{Imax}-\left(\gamma +k_{T}\right)k_{V}}$$

$$=\frac{c/\left(\beta E\right)}{\;\left(\gamma -\beta \left(\gamma +k_{T}\right)\right)e^{\beta \int_{0}^{t_{Imax}}S\left(\tau \right)d\tau }e^{-\left(\gamma +\;k_{T}\right)t_{Imax}}I_{0}-\left(\gamma +k_{T}\right)k_{V}}>0$$

Property (v) has been proved. Property (vi) is obvious by zeroing (26). □

**Figure 1:**
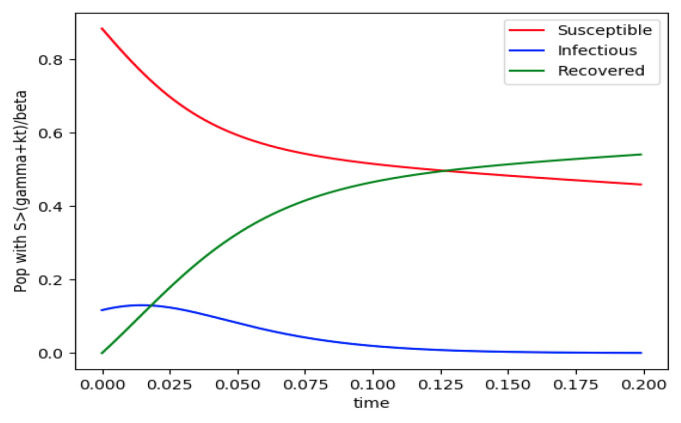
$N_{0}=1$ and the initial conditions constraints of Theorem 4 hold with ${\dot{I}}_{0}>0$.

**Figure 2:**
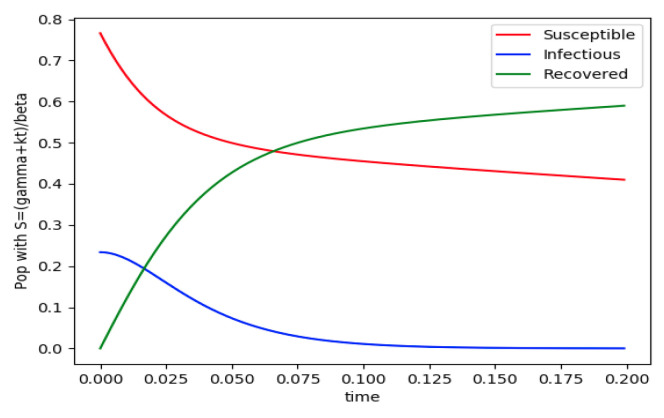
$N_{0}=1$ and the initial conditions constraints of Theorem 4 fail with ${\dot{I}}_{0}=0$.

**Figure 3:**
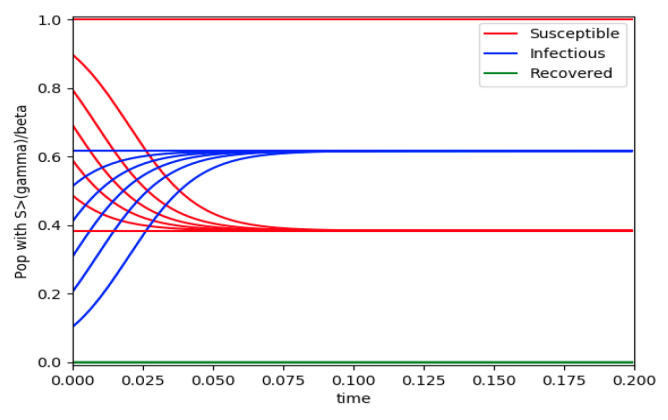
$N_{0}=1$ and the initial conditions constraints of Theorem 4 hold with no controls used.

**Figure 4:**
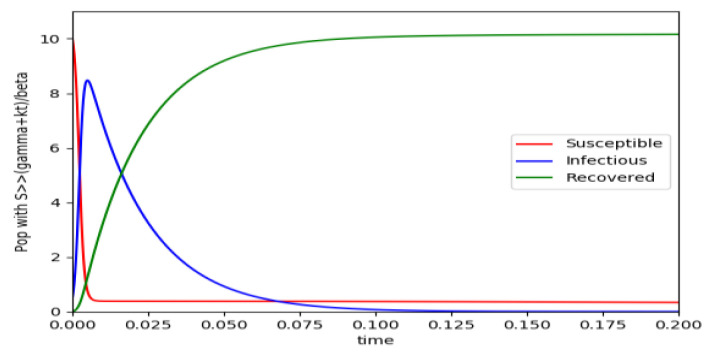
$S_{0}>1,\;I_{0}>1$ (unnormalized to unity total population) and the initial conditions constraints of Theorem 4 hold with ${\dot{I}}_{0}>0$.

Example 4 is tested numerically in the sequel with the following data *β* = 30, *γ* = 50 years^−1^, implying that the average infectious period is *T_γ_* = 365/50 = 7.3 days, *k_V_* = 1 and *k_T_* = 50. The time scale of the figures is in a scale of years accordingly with the above numerical values. In Figure 1, the solution trajectories of all the subpopulation are shown with the constraints of Theorem 4 being fulfilled by the initial conditions, in particular $S_{0}>\;\frac{\gamma +k_{T}}{\beta }$, $I_{0}=1-S_{0}$ and $R_{0}=0$ so that $N_{0}$ is normalized to unity. It is seen that the infectious subpopulation trajectory has a maximum at a finite time and that the state trajectory solution converges asymptotically to an endemic equilibrium point. In Figure 2, the state trajectory solution is shown with $N_{0}=1$ when $S_{0}=\left(\gamma +k_{T}\right)/\beta$ which violates the conditions of Theorem 4 with ${\dot{I}}_{0}=0$. In this case, there is no relative maximum of the infectious subpopulation at finite time. In both situations, it has been observed by extending the overall simulation time that the susceptible and the infectious subpopulations converge asymptotically to zero while the recovered subpopulation converges to unity as time tends to infinity. The controls are suppressed in Figure 3 with *N*_0_ = 1. In this case, the recovered subpopulation may be deleted from the model since it is unnecessary while being identically zero. The infectious and susceptible subpopulations are in an endemic equilibrium point for all time so that the infection results to be permanent in the sense that it cannot be asymptotically removed. See Theorem 4(vi) for the case *k_V_*= 0. Figure 4 exhibits a trajectory solution which agrees with Theorem 4 while there is no normalization of the initial conditions to unity. In this case, the maximum of the infectious subpopulation at a finite time becomes very apparent.

## 4. Links with Entropy and Maximum Dissipation Mechanism Issues

### 4.1. Comparison of the Epidemic Model and Reference Model Information Entropies

Since (1) is a scalar equation, a valid solution for the particular model-dependent time-varying coefficient α(t)=−cln(g(t)/E)/h(t) of Theorem 2 and Theorem 3 is, according to Theorem 1:

(32) $$I\left(t\right)=e^{-c\int_{0}^{t}h^{-1}\left(\tau \right)\;ln\left(g\left(\tau \right)/E\right)d\tau }I_{0};\;t\in R_{0+}$$

Under the particular constraints E=D, $c=\left(1-ln\left(L/D\right)\right)/{ln}^{2}\left(L/D\right)$ and g(t)=h(t)=t, it is got in [11] that $\alpha \left(t\right)={\left[\left(ln\left(L/D\right)-1\right)/{ln}^{2}\left(L/D\right)\right]}^{}t^{-1}ln\left(t/D\right)$ and (32), namely:

(33) $$I_{p}\left(t\right)=e^{\left(ln\left(L/D\right)-1\right)/{ln}^{2}\left(L/D\right)\int_{0}^{t}\tau^{-1}ln\left(\tau /D\right)d\tau }I_{0};\;t\in R_{+}$$

approaches the log-normal distribution:

(34) $$I_{r}\left(t\right)=\frac{k}{\sqrt{2\pi }\;\sigma_{r}t}e^{-\frac{{\left(ln\;t-\mu_{r}\right)}^{2}}{2\sigma_{r}^{2}}};\;t\in R_{+}$$

for reference values $D=D_{r}$ and $L=L_{r}$ of the maximum and inflection reference time instants where $\mu_{r}=lnD_{r}+\sigma_{r}^{2}$ and $\sigma_{r}$ is given by the principle of extreme entropy production rate, typically $\sigma_{r}\approx 0.408$ gives the width of the distribution function for the maximum dissipation rate for the usual definition of the Shannon entropy. The main reason for the limitation of such a width is that the medical and social interventions are a dissipation mechanism which controls and limits the disease propagation. Comparing (33) and (34), one gets that $k=\sqrt{2\pi }\;\sigma_{r}^{3}I_{0}$ after solving the indetermination 0/0 at t=0 via L´ $H\hat{o}\mathrm{pital}$ rule leading to the “infection reference evolution” $I_{r}\left(t\right)=I_{p}\left(t\right)$, that is by equalizing (23) and (24), under the above set of particular constraints, where:

(35) $$I_{r}\left(t\right)=t^{-1}\sigma_{r}^{2}e^{-\frac{{\left(lnt-lnD_{r}-\sigma_{r}^{2}\right)}^{2}}{2\sigma_{r}^{2}}}I_{0};\;t\in R_{+}$$

Now, equalize $I\left(t\right)=I_{r}\left(t\right)+\tilde{I}\left(t\right)$; $\forall t\in R_{+}$ for some perturbation function $\tilde{I}:R_{+}\to R_{0+}$ resulting to be from (32) and (35) for $I_{0}>0$:

(36) $$\tilde{I}\left(t\right)=\left(e^{-c\int_{0}^{t}h^{-1}\left(\tau \right)\;ln\left(g\left(\tau \right)/E\right)d\tau }-t^{-1}\sigma_{r}^{2}e^{-\frac{{\left(lnt-lnD_{r}-\sigma_{r}^{2}\right)}^{2}}{2\sigma_{r}^{2}}}\right)I_{0};\;t\in R_{+}$$

The Shannon entropy of the infection $S_{I}\left(\eta \right)$ results to be given by the following Riemann- Stieljes integral which quantifies the entropy error ${\tilde{S}}_{I}\left(\eta \right)$ of that associated with any given model related to the entropy of the “infection reference evolution” given by the log- normal function $S_{I_{r}}\left(\eta \right)=S_{I_{r}}\left(\eta ,\sigma_{r}\right)$ for the given reference width value $\sigma_{r}=\sqrt{1/2\eta }$:

(37) $$\begin{array}{c} S_{I}\left(\eta \right)=-\int_{0}^{\infty }t^{1-\eta }I\left(t\right)ln\left(t^{1-\eta }I\left(t\right)\right)dt^{\eta }\\ =-\int_{0}^{\infty }t^{1-\eta }I\left(t\right)\left(\left(1-\eta \right)lnt+lnI\left(t\right)\right)dt^{\eta }\\ =-\int_{0}^{\infty }t^{1-\eta }\left(I_{r}\left(t\right)+\tilde{I}\left(t\right)\right)\left(\left(1-\eta \right)lnt+ln\left(I_{r}\left(t\right)\left(1+I_{r}^{-1}\left(t\right)\tilde{I}\left(t\right)\right)\right)\right)dt^{\eta }\\ =-\int_{0}^{\infty }t^{1-\eta }\left(I_{r}\left(t\right)+\tilde{I}\left(t\right)\right)\left(\left(1-\eta \right)lnt+lnI_{r}\left(t\right)+ln\left(1+I_{r}^{-1}\left(t\right)\tilde{I}\left(t\right)\right)\right)dt^{\eta }\\ =S_{I_{r}}\left(\eta \right)-\int_{0}^{\infty }t^{1-\eta }I_{r}\left(t\right)ln\left(1+I_{r}^{-1}\left(t\right)\tilde{I}\left(t\right)\right)dt^{\eta }-\int_{0}^{\infty }t^{1-\eta }\tilde{I}\left(t\right)lnI\left(t\right)dt^{\eta }-\left(1-\eta \right)\int_{0}^{\infty }t^{1-\eta }\tilde{I}\left(t\right)lntdt^{\eta }\\ =S_{I_{r}}\left(\eta \right)-\int_{0}^{\infty }t^{1-\eta }I_{r}\left(t\right)ln\left(1+I_{r}^{-1}\left(t\right)\tilde{I}\left(t\right)\right)dt^{\eta }-\left(1-\eta \right)\int_{0}^{\infty }t^{1-\eta }\tilde{I}\left(t\right)lntdt^{\eta }\\ +\int_{0}^{\infty }t^{1-\eta }I_{r}\left(t\right)\left(1-I_{r}^{-1}\left(t\right)I\left(t\right)\right)lnI\left(t\right)dt^{\eta }\\ =S_{I_{r}}\left(\eta \right)+{\tilde{S}}_{I}\left(\sigma \right);\;t\in R_{+} \end{array}$$

after using $I\left(t\right)=I_{r}\left(t\right)\;\left(1+I_{r}^{-1}\left(t\right)\tilde{I}\left(t\right)\right)$ and its equivalent expression $\tilde{I}\left(t\right)=-I_{r}\left(t\right)\left(1-I_{r}^{-1}\left(t\right)I\left(t\right)\right)$, where the reference entropy based on the identification of the log-normal function (34)with the solution of (1), that is, (33), yields for $\sigma_{r}=\sqrt{1/2\eta }$:

(38) $$\begin{array}{c} S_{I_{r}}\left(\;\eta \right)=-\int_{0}^{\infty }t^{1-\eta }I_{r}\left(t\right)ln\left(t^{1-\eta }I_{r}\left(t\right)\right)dt^{\eta }\\ =\eta \left(ln\left(\sqrt{\frac{\pi }{\eta }}\right)+\eta \left(lnD_{r}+\frac{1}{2\eta }\right)+\frac{1}{2}\right) \end{array}$$

after converting the Riemann-Stieljes integral (39) in a Riemann integral via differentiation of $dt^{\eta }$ by using (35). Note that it is assumed that both current and reference entropies are evaluated for the same parameter η which is typically chosen as η=3. At the same time, it is assumed that the maximum dissipation rate proportional to the maximum rate of entropy production is governed by the width of the distribution function σ. So the current model can potentially have a value $\sigma \neq \sigma_{r}$. See [11] for the normalized case obtained for $I_{0}=1$, and, also one gets the following entropy error:

(39) $$\begin{array}{c} {\tilde{S}}_{I}\left(\eta \right)=-\int_{0}^{\infty }t^{1-\eta }{\left[ln\left({\left(I\left(t\right)/I_{r}\left(t\right)\right)}^{I_{r}\left(t\right)}\right)+ln\left(I{\left(t\right)}^{I\left(t\right)-I_{r}\left(t\right)}\right)\right]}^{}dt^{\eta }-\left(1-\eta \right)\int_{0}^{\infty }t^{1-\eta }\tilde{I}\left(t\right)lntdt^{\eta }\\ =-\int_{0}^{\infty }t^{1-\eta }{\left[ln\left({\left(I\left(t\right)/I_{r}\left(t\right)\right)}^{I_{r}\left(t\right)}\left(I{\left(t\right)}^{I\left(t\right)-I_{r}\left(t\right)}\right)\right)\right]}^{}dt^{\eta }+\left(1-\eta \right)\int_{0}^{\infty }t^{1-\eta }I_{r}\left(t\right)\left(1-I_{r}^{-1}\left(t\right)I\left(t\right)\right)lntdt^{\eta }\\ =-\eta \int_{0}^{\infty }ln\left(\frac{I{\left(t\right)}^{I\left(t\right)}}{I_{r}{\left(t\right)}^{I_{r}\left(t\right)}}\right)dt+\eta \left(\eta -1\right)\int_{0}^{\infty }ln\left(t^{I_{r}\left(t\right)-I\left(t\right)}\right)dt\\ =-\eta \int_{0}^{\infty }ln\left(\frac{I{\left(t\right)}^{I\left(t\right)}}{I_{r}{\left(t\right)}^{I_{r}\left(t\right)}t^{\left(\eta -1\right)\left(I_{r}\left(t\right)-I\left(t\right)\right)}}\right)dt;\;t\in R_{+} \end{array}$$

It turns out obvious that the integrand of (39) is identically zero if $\tilde{I}\left(t\right)\equiv 0$, so that $I\left(t\right)\equiv I_{r}\left(t\right)$, leading to ${\tilde{S}}_{I}\left(\eta \right)\equiv 0$. The expression (37), subject to (38)–(39), parameterizes the incremental entropy with the same parameter η which parameterizes the reference entropy $S_{I_{r}}\left(\;\eta_{r}\right)$. Now, define the error:

(40) $$\delta \left(t\right)=\frac{I{\left(t\right)}^{I\left(t\right)}}{I_{r}{\left(t\right)}^{I_{r}\left(t\right)}t^{\left(\eta -1\right)\left(I_{r}\left(t\right)-I\left(t\right)\right)}}-1;\;t\in R_{0+}$$

so that ${\tilde{S}}_{I}\left(\eta \right)\equiv 0$ if δ(t)≡0 and, expanding $ln\left(\frac{I{\left(t\right)}^{I\left(t\right)}}{I_{r}{\left(t\right)}^{I_{r}\left(t\right)}}\right)$ via the Newton- Mercator series for the logarithm, leads to:

(41) $$ln\left(\frac{I{\left(t\right)}^{I\left(t\right)}}{I_{r}{\left(t\right)}^{I_{r}\left(t\right)}t^{\left(\eta -1\right)\left(I_{r}\left(t\right)-I\left(t\right)\right)}}\right)=ln\left(1+\delta \left(t\right)\right)=\delta \left(t\right)+\sum_{n=2}^{\infty }\frac{{\left(-1\right)}^{n+1}}{n}\;\delta^{n}\left(t\right);\;t\in R_{0+}$$

and such a series converges to ln(1+δ(t)) for all $t\in R_{0+}$ provided that δ(t)∈(−1, 1], equivalently, $\tilde{I}\left(t\right)\in \left(-I_{r}\left(t\right),\;I_{r}\left(t\right)\right]$; $\forall t\in R_{0+}$; $\forall t\in R_{0+}$. Thus, the following description in linear and higher-order additive terms of the entropy error follows from (40)–(41) into (39):

(42) $${\tilde{S}}_{I}\left(\eta \right)={\tilde{S}}_{IL}\left(\eta \right)+{\tilde{\tilde{S}}}_{I}\left(\eta \right);\;t\in R_{0+}$$

where:

(43) $${\tilde{S}}_{IL}\left(\eta \right)=-\eta \int_{0}^{\infty }\left(\frac{I{\left(t\right)}^{I\left(t\right)}}{I_{r}{\left(t\right)}^{I_{r}\left(t\right)}t^{\left(\eta -1\right)\left(I_{r}\left(t\right)-I\left(t\right)\right)}}-1\right)dt;\;t\in R_{0+}$$

(44) $${\tilde{\tilde{S}}}_{I}\left(\eta \right)=-\eta \left(\sum_{n=2}^{\infty }\frac{{\left(-1\right)}^{n+1}}{n}\;{\left(\frac{I{\left(t\right)}^{I\left(t\right)}}{I_{r}{\left(t\right)}^{I_{r}\left(t\right)}t^{\left(\eta -1\right)\left(I_{r}\left(t\right)-I\left(t\right)\right)}}-1\right)}^{n}dt\right);\;t\in R_{0+}$$

The subsequent results hold related to the case when the error between the infectious functions of the model and the reference one associated to the log-normal function converges asymptotically to zero as time tends to infinity. The first result, stated separately by convenience concerned its proof, discusses the simplest case for η=1.

**Proposition** **1.** *Assume that* η=1 *and* $\underset{t\to +\infty \;}{lim}\left|\int_{0}^{t}ln\left(\frac{I{\left(\tau \right)}^{I\left(\tau \right)}}{I_{r}{\left(\tau \right)}^{I_{r}\left(\tau \right)}}\right)d\tau \right|<+\infty$ *.*

*then,* ${\tilde{S}}_{I}\left(\;1\right)<+\infty$ *for all* $t\in R_{0+}$ *and* $\underset{t\to +\infty }{lim}\left(I\left(t\right)-I_{r}\left(t\right)\right)=0$ *.*

**Proof.**  Note from (39) that ${\tilde{S}}_{I}\left(1\right)=\int_{0}^{\infty }ln\left(\frac{I{\left(t\right)}^{I\left(t\right)}}{I_{r}{\left(t\right)}^{I_{r}\left(t\right)}}\right)dt<+\infty$. Since the function $\frac{I{\left(t\right)}^{I\left(t\right)}}{I_{r}{\left(t\right)}^{I_{r}\left(t\right)}}$ is uniformly continuous on $R_{0+}$ and $\underset{t\to +\infty \;}{lim}\left|\int_{0}^{t}ln\left(\frac{I{\left(\tau \right)}^{I\left(\tau \right)}}{I_{r}{\left(\tau \right)}^{I_{r}\left(\tau \right)}}\right)d\tau \right|<+\infty$ then $ln\frac{I{\left(t\right)}^{I\left(t\right)}}{I_{r}{\left(t\right)}^{I_{r}\left(t\right)}}\to 0$ as t→+∞ from Barbalat´s lemma and then $\frac{I{\left(t\right)}^{I\left(t\right)}}{I_{r}{\left(t\right)}^{I_{r}\left(t\right)}}\to 1$ as t→+∞. It is clear that a limit solution which satisfies this constraint is $\underset{t\to +\infty }{lim}\left(I\left(t\right)-I_{r}\left(t\right)\right)=0$. It is now proved that no alternative limiting constraint on the pair $\left(I\left(t\right),\;I_{r}\left(t\right)\right)$ as t→+∞ is compatible with $\underset{t\to +\infty }{lim}\frac{I{\left(t\right)}^{I\left(t\right)}}{I_{r}{\left(t\right)}^{I_{r}\left(t\right)}}=1$. Assume that $\underset{t\to +\infty }{lim\;\inf}\left|\;I\left(t\right)-I_{r}\left(t\right)\right|>0$ It can happen that:

(a) $\underset{t\to +\infty }{lim\;\inf}\left(I\left(t\right)-I_{r}\left(t\right)\right)>0$. Then, $\underset{t\to +\infty }{lim\;\inf}ln\frac{I{\left(t\right)}^{I\left(t\right)}}{I_{r}{\left(t\right)}^{I_{r}\left(t\right)}}=\underset{t\to +\infty }{lim\;\inf}\left(I\left(t\right)lnI\left(t\right)-I_{r}\left(t\right)lnI_{r}\left(t\right)\right)$$>\underset{t\to +\infty }{lim\;\inf}\left(I_{r}\left(t\right)lnI\left(t\right)-I_{r}\left(t\right)lnI_{r}\left(t\right)\right)>\underset{t\to +\infty }{lim\;\inf}\left(I_{r}\left(t\right)lnI_{r}\left(t\right)-I_{r}\left(t\right)lnI_{r}\left(t\right)\right)=0$ so that $\underset{t\to +\infty }{lim\;\inf}ln\frac{I{\left(t\right)}^{I\left(t\right)}}{I_{r}{\left(t\right)}^{I_{r}\left(t\right)}}>0$. Hence, a contradiction to Barbalat´s lemma; or
(b) $\underset{t\to +\infty }{lim\;\inf}\left(I_{r}\left(t\right)-I\left(t\right)\right)>0$. Under a similar reasoning to that of a), one gets that $\underset{t\to +\infty }{lim\;\inf}ln\frac{I_{r}{\left(t\right)}^{I_{r}\left(t\right)}}{I{\left(t\right)}^{I\left(t\right)}}>0$. Again, a contradiction to Barbalat´s lemma.

The second result discusses the simplest case for η≠1. It is seen that the basic limit result $\underset{t\to +\infty }{lim}\left(I\left(t\right)-I_{r}\left(t\right)\right)=0$ of Proposition 1 is still kept under the reasonable assumption that the infection and reference infection functions are bounded. □

**Proposition** **2.** *Assume that* η≠1 *,* $I,I_{r}:\;R_{0+}\to R_{0+}$ *are bounded and* $\underset{t\to +\infty \;}{lim}\left|\int_{0}^{t}ln\left(\frac{I{\left(\tau \right)}^{I\left(\tau \right)}}{I_{r}{\left(\tau \right)}^{I_{r}\tau }t^{\left(\eta -1\right)\left(I_{r}\left(\varsigma \right)-I\left(\tau \right)\right)}}\right)d\tau \right|<+\infty$ *.*

*Then,* ${\tilde{S}}_{I}\left(\;\eta \right)<+\infty$ *for all* $t\in R_{0+}$ *and* $\underset{t\to +\infty }{lim}\left(I\left(t\right)-I_{r}\left(t\right)\right)=0$ *.*

**Proof.**  Note that ${\tilde{S}}_{I}\left(1\right)<+\infty$ and that, from the uniform continuity of $\frac{I{\left(t\right)}^{I\left(t\right)}}{I_{r}{\left(t\right)}^{I_{r}\left(t\right)}t^{\left(\eta -1\right)\left(I_{r}\left(t\right)-I\left(t\right)\right)}}$ everywhere in $R_{0+}$, the boundedness of its integral on [0,∞) and Barbalat´s lemma, it follows that $\frac{I{\left(t\right)}^{I\left(t\right)}}{I_{r}{\left(t\right)}^{I_{r}\left(t\right)}t^{\left(\eta -1\right)\left(I_{r}\left(t\right)-I\left(t\right)\right)}}\to 1$ as t→+∞ what implies that:

$$\underset{t\to +\infty }{lim}\left(I\left(t\right)lnI\left(t\right)-I_{r}\left(t\right)lnI_{r}\left(t\right)+\left(1-\eta \right)\left(I_{r}\left(t\right)-I\left(t\right)\right)\;ln\;t\right)=0$$

If η>1 and lnt→∞ as t→∞ then there exists some strictly increasing real sequence ${\left\{t_{i}\right\}}_{i=0}^{\infty }$, such that $\underset{k\to \infty }{lim}\left|\left(1-\eta \right)\left(I_{r}\left(\left(t_{k}\right)\right)-I\left(\left(t_{k}\right)\right)\right)\;ln\;t_{k}\right|=\infty$ with $t_{k}\in {\left\{t_{i}\right\}}_{i=0}^{\infty }$ if $\underset{t\to +\infty }{lim}\left(I\left(t\right)-I_{r}\left(t\right)\right)\neq 0$. But this can hold only if $\underset{k\to +\infty }{lim}\left|\;I\left(t_{k}\right)lnI\left(t_{k}\right)-I_{r}\left(t_{k}\right)lnI_{r}\left(t_{k}\right)\right|=+\infty$. But, since $I_{r}:\;R_{0+}\to R_{0+}$ is bounded for all time, this implies that $I\left(t_{k}\right)\to +\infty$ as $t_{k}\left(\in {\left\{t_{i}\right\}}_{i=0}^{\infty }\right)\to +\infty$ and $I:\;R_{0+}\to R_{0+}$ is unbounded. But then

$$\underset{k\to +\infty }{lim}\left(I\left(t_{k}\right)lnI\left(t_{k}\right)-I_{r}\left(t_{k}\right)lnI_{r}\left(t_{k}\right)+\left(\eta -1\right)\left(I\left(t_{k}\right)-I_{r}\left(t_{k}\right)\right)\;ln\;t_{k}\right)=\infty +\infty =\infty$$

and a contradiction follows to the above limit to be zero. As a result, $\underset{t\to +\infty }{lim}\left(I\left(t\right)-I_{r}\left(t\right)\right)=0$ if η>1.

Now, assume that η<1. Since $\underset{k\to \infty }{lim}\left|\left(1-\eta \right)\left(I_{r}\left(t_{k}\right)-I\left(t_{k}\right)\right)\;ln\;t_{k}\right|=\infty$ for $t_{k}\left(\in {\left\{t_{i}\right\}}_{i=0}^{\infty }\right)\to +\infty$ and some strictly increasing real sequence ${\left\{t_{i}\right\}}_{i=0}^{\infty }$, provided that $\underset{t\to +\infty }{lim}\left(I\left(t\right)-I_{r}\left(t\right)\right)\neq 0$, then $I\left(t_{k}\right)\to +\infty$ as $t_{k}\left(\in {\left\{t_{i}\right\}}_{i=0}^{\infty }\right)\to +\infty$ since $I_{r}:\;R_{0+}\to R_{0+}$ is bounded. Since $I:\;R_{0+}\to R_{0+}$ is unbounded, because it has a divergent subsequence ${\left\{I\left(t_{k}\right)\right\}}_{k=0}^{\infty }$ and it is a solution of a unstable time-invariant linear differential system, it is of positive exponential order $\varsigma_{0}>0$ and there exists a real constant $\varsigma <\varsigma_{0}$ such that $I\left(t_{k}\right)\geq e^{\varsigma t_{k}}$; $\forall t_{k}\in {\left\{t_{i}\right\}}_{i=0}^{\infty }$ and $I\left(t_{k}\right)/lnt_{k}\left(\geq e^{\varsigma t_{k}}/lnt_{k}\right)\to \infty$ as $t_{k}\left(\in {\left\{t_{i}\right\}}_{i=0}^{\infty }\right)\to \infty$ and, furthermore,

$$\underset{k\to +\infty }{lim}\left(I\left(t_{k}\right)lnI\left(t_{k}\right)-I_{r}\left(t_{k}\right)lnI_{r}\left(t_{k}\right)\right)=\left(1-\eta \right)\underset{k\to +\infty }{lim}\left(I\left(t_{k}\right)-I_{r}\left(t_{k}\right)\right)\;ln\;t_{k}=\infty$$

but the expression below is an infinity limit (and not a ∞−∞ indetermination since $I\left(t_{k}\right)/lnt_{k}\to \infty$):

$$\underset{k\to +\infty }{lim}\left(I\left(t_{k}\right)lnI\left(t_{k}\right)-I_{r}\left(t_{k}\right)lnI_{r}\left(t_{k}\right)-\left(1-\eta \right)\left(I\left(t_{k}\right)-I_{r}\left(t_{k}\right)\right)\;ln\;t_{k}\right)=\infty$$

which contradicts:

$$\underset{t\to +\infty }{lim}\left(I\left(t\right)lnI\left(t\right)-I_{r}\left(t\right)lnI_{r}\left(t\right)+\left(1-\eta \right)\left(I_{r}\left(t\right)-I\left(t\right)\right)\;ln\;t\right)=0$$

As a result, $\underset{t\to +\infty }{lim}\left(I\left(t\right)-I_{r}\left(t\right)\right)=0$ if η≠1.

It is now briefly discussed the fact that the boundedness hypothesis of Proposition 2 is not very restrictive for some of the given examples, like for instance, Examples 2,3, where the infectious subpopulation converges asymptotically to zero. For such a purpose, note from (35) that $I_{r}\left(t\right)\to 0$ exponentially fast as t→∞. In example 2, I(t)→0 exponentially as t→∞ so their difference function also converges to zero exponentially as t→∞. The integral boundedness invoked in the assumption of Proposition 2 is of the form $F=\left|\;\int_{0}^{\infty }ln\;x\left(t\right)dt\right|<+\infty$, where $x\left(t\right)=\frac{I{\left(t\right)}^{I\left(t\right)}}{I_{r}{\left(t\right)}^{I_{r}\left(t\right)}t^{\left(\eta -1\right)\left(I_{r}t-I\left(t\right)\right)}}$ is everywhere differentiable with respect to time. In order to convert the elevant Riemann-Stieljes integral into a standard Riemann one, take $dx=\dot{x}\left(t\right)dt$ and, later on, perform the change of variable x→u defined by u=lnx, du=dx/x to yield:

$$F=\left|\;\int_{x_{0}}^{1}\frac{ln\;x}{\dot{x}}dx\right|=\left|\;\int_{x_{0}}^{1}\frac{ln\;x}{x}\frac{x}{\dot{x}}dx\right|\leq \left(\underset{0\leq t\leq +\infty }{sup}\left|\frac{x\left(t\right)}{\dot{x}\left(t\right)}\right|\right)\left|\;\int_{x_{0}}^{1}\frac{ln\;x}{x}dx\right|$$

$$=M\left(\eta \right)\left|\;\int_{lnx_{0}}^{0}udu\right|=M\left(\eta \right)\frac{lnx_{0}^{2}}{2}$$

where $x\left(0\right)=x_{0}$ and $M\left(\eta \right)=\underset{0\leq t\leq +\infty }{sup}\left|\frac{x\left(t\right)}{\dot{x}\left(t\right)}\right|\leq +\infty$ for the given constant η. Note that M(η)<+∞ if and only if the set of zeros of $\dot{x}\left(t\right)$ at any finite time instant is empty, that is, if and only if $Z_{xdot}\left(\eta \right)=\emptyset$, where $Z_{xdot}\left(\eta \right)=\left\{t\geq 0\;:\;\dot{x}\left(t\right)=0\;\right\}=\emptyset$ (equivalently, M(η)=+∞ if and only if $Z_{xdot}\left(\eta \right)\neq \emptyset$. Rewriting $x\left(t\right)=\frac{y\left(t\right)}{t^{\eta -1}z\left(t\right)}$ it follows that $\dot{x}\left(t\right)=0$ for any t≥0 if and only the following constraint holds $t=\frac{\left(\eta -1\right)z\left(t\right)y\left(t\right)}{z\left(t\right)\dot{y}\left(t\right)-y\left(t\right)\dot{z}\left(t\right)}$. Therefore, $Z_{xdot}\left(\eta \right)=\left\{t\geq 0\;:\;t=\frac{\left(\eta -1\right)z\left(t\right)y\left(t\right)}{z\left(t\right)\dot{y}\left(t\right)-y\left(t\right)\dot{z}\left(t\right)}\;\right\}\neq \emptyset$ is an event of zero probability. Thus, the boundedness hypothesis of Proposition 2 happens almost surely in the event that the infectious subpopulation converges asymptotically to zero as time tends to infinity. □

Propositions 1 and 2 yield the direct joint result independently of the value of η:

**Proposition** **3.** *Assume that* $\eta \in R_{0+}$ *,* $I,I_{r}:\;R_{0+}\to R_{0+}$ *are bounded and* $\underset{t\to +\infty \;}{lim}\left|\int_{0}^{t}ln\left(\frac{I{\left(t\right)}^{I\left(t\right)}}{I_{r}{\left(t\right)}^{I_{r}\left(t\right)}t^{\left(\eta -1\right)\left(I_{r}\left(t\right)-I\left(t\right)\right)}}\right)dt\right|<+\infty$ *.*

*Then,* ${\tilde{S}}_{I}\left(\;\eta \right)<+\infty$ *for all* $t\in R_{0+}$ *and* $\underset{t\to +\infty }{lim}\left(I\left(t\right)-I_{r}\left(t\right)\right)=0$ *.*

Concerning Proposition 3, note that the boundedness of ${\tilde{S}}_{I}\left(\;\eta \right)$ does not guarantee that the linear part and the remaining part of higher- order terms in the decomposition of (42), subject to (43) and (44), are both finite. It could “a priori” happen that they both tend to infinity with opposite signs. But if any of them is bounded, the other one should be bounded as well according to Proposition 3. Fortunately, this does not happen under weak extra assumptions. In particular, the following result holds:

**Proposition** **4.** *Assume that* $\eta \in R_{0+}$ *,* $I,I_{r}:\;R_{0+}\to R_{0+}$ *are bounded, and*

$$\int_{0}^{\infty }ln\left(\frac{I{\left(t\right)}^{I\left(t\right)}}{I_{r}{\left(t\right)}^{I_{r}\left(t\right)}t^{\left(\eta -1\right)\left(I_{r}\left(t\right)-I\left(t\right)\right)}}-1\right)dt<+\infty .$$

*Then,* ${\tilde{S}}_{IL}\left(\eta \right)<+\infty$ *;* $t\in R_{0+}$ *.*

*If, in addition,* $\int_{0}^{\infty }ln\left(\frac{I{\left(t\right)}^{I\left(t\right)}}{I_{r}{\left(t\right)}^{I_{r}\left(t\right)}t^{\left(\eta -1\right)\left(I_{r}\left(t\right)-I\left(t\right)\right)}}dt\right)<+\infty$ *then* $\left|{\tilde{\tilde{S}}}_{I}\left(\eta \right)\right|<+\infty$ *and* ${\tilde{S}}_{I}\left(\eta \right)<+\infty$ *for all* $t\in R_{0+}$ *and* $\underset{t\to +\infty }{lim}\left(I\left(t\right)-I_{r}\left(t\right)\right)=0$ *.*

**Proof.**  It is direct to see that ${\tilde{S}}_{IL}\left(\eta \right)<+\infty$. Also, and again from Barbalat´s lemma, $\frac{I{\left(t\right)}^{I\left(t\right)}}{I_{r}{\left(t\right)}^{I_{r}\left(t\right)}t^{\left(\eta -1\right)\left(I_{r}\left(t\right)-I\left(t\right)\right)}}\to 1$ as t→+∞. Thus, from Proposition 3, $\underset{t\to +\infty }{lim}\left(I\left(t\right)-I_{r}\left(t\right)\right)=0$. If, furthermore, $\int_{0}^{\infty }ln\left(\frac{I{\left(t\right)}^{I\left(t\right)}}{I_{r}{\left(t\right)}^{I_{r}\left(t\right)}t^{\left(\eta -1\right)\left(I_{r}\left(t\right)-I\left(t\right)\right)}}\right)dt<+\infty$ then, again from Proposition 3, ${\tilde{S}}_{I}\left(\eta \right)<+\infty$ and $-\infty <-\left|\tilde{S}\left(\eta \right)-{\tilde{S}}_{IL}\left(\eta \right)\right|\leq {\tilde{\tilde{S}}}_{I}\left(\eta \right)\leq \left|\tilde{S}\left(\eta \right)-{\tilde{S}}_{IL}\left(\eta \right)\right|<+\infty$. □

Note that the above results agree with the asymptotic results of Examples 1–4, where I(t)→0 as t→∞, and with Theorem 1, since the reference $I_{r}\left(t\right)\to 0$, jointly implying $\left(I\left(t\right)-I_{r}\left(t\right)\right)\to 0$ as t→∞.

**Remark** **4.** *The rationale behind the definition of a time-varying coefficient in (1) is to reduce the higher-order epidemic model with two or more states to a single-order differential equation based on the assumption that the log-normal distribution is a sufficiently accurate model for the infectious evolution. It is apparent that the profile of the log-normal distribution remembers the behavior of the strong infections in their blowing–up evolution phase along time. However, it is obvious that the epidemic models have the concourse of several coupled subpopulations so that it the model is reduced to a first-order dynamics the influence of the remaining dynamics should be accounted for through a time-varying parameterization and dynamics uncertainty in (1) since the model order is reduced to unity. The accuracy of the modeling procedure is evaluated by means of the entropy through (37). Hence if the actual infectious population curve is close to the reference one, then we have* $S_{I}\left(\eta \right)=S_{I_{r}}\left(\eta \right)$ *which generates the dissipation rate of the model. On the other hand, if the current system differs from the reference model, then the entropy becomes corrected with the additional term* ${\tilde{S}}_{I}\left(\eta \right)$ *. Therefore, the contributing terms in (37) provide an estimation of the modeling uncertainty based on the assumed log- normal reference distribution. As a result, the best approximation of the current model to the reference one is that which minimizes the error entropy* ${\tilde{S}}_{I}\left(\eta \right)$ *, i.e., the one which reduces as much as possible the uncertainty introduced by the approximation.*

**Remark** **5.** *Note that the entropy of the infection* I(t) *for* η=1 *is defined as* $S_{I}\left(1\right)=-\int_{0}^{\infty }I\left(\tau \right)lnI\left(\tau \right)d\tau$ *The entropy of the truncated function* $I_{t}\left(\tau \right)=I\left(\tau \right)$ *for* τ∈[0,t] *and* $I_{t}\left(\tau \right)=0$ *for* τ∉[0,t] *is* $S_{I_{t}}\left(1\right)=-{\int_{0}^{\infty }I}_{t}\left(\tau \right)lnI_{t}\left(\tau \right)d\tau =-\int_{0}^{t}I\left(\tau \right)lnI\left(\tau \right)d\tau$ *. Note also that* ${\dot{S}}_{I_{t}}\left(1\right)=-I\left(t\right)lnI\left(t\right)$ *and* ${\ddot{S}}_{I_{t}}\left(1\right)=-\dot{I}\left(t\right)\left(1+lnI\left(t\right)\right)=0$ *if* t=D *. That is, the inflection point of the truncated entropy occurs at the relative extreme values of* I(t) *. In particular, if the infection is in its first expanding phase, this occurs at its maximum* t=D *.*

### 4.2. Estimation of Errors of the Distribution Widths between the Log-Normal Reference and Current Model Information Entropies

One gets from (38) for the usual reference entropy definition based on the log-normal distribution of width $\sigma_{r}=\frac{1}{\sqrt{2\eta }}$, [11,33,37], that:

(45) $$S_{I_{r}}\left(\sigma_{r},D_{r},\eta \right)=\eta \left(ln\left(\sqrt{2\pi }\sigma_{r}\right)+\eta \left(lnD_{r}+\sigma_{r}^{2}\right)+\frac{1}{2}\right)$$

and the particular value:

(46) $$\sigma_{r}=\sigma_{r}\left(\eta \right)=\arg\left(r\in R_{0+}\;:\;\frac{d^{2}S\left(\sigma_{r},D_{r},\eta \right)}{dr^{2}}=0\right)$$

is the width distribution maximum value which makes the reference entropy to cease to increase while giving the maximum dissipation rate which leads to:

(47) $$S_{I_{r}}\left(\sqrt{\frac{1}{2\eta }},\;D_{r},\eta \right)=\eta \left(ln\sqrt{\frac{\pi }{\eta }}+\eta \left(lnD_{r}+\sigma_{r}^{2}\right)+\frac{1}{2}\right)=\arg\;\left(S_{I_{r}}\left(\sigma_{r},\;\eta \right)\;:\;\frac{d^{2}S\left(\sigma_{r},\eta \right)}{d\sigma_{r}^{2}}=0\right)$$

Note that the above reference description is easily associated to an epidemic model given by a first-order differential equation involving only the infection evolution. Note also, in particular, that the infection curve solution is of exponential order as it is the log-normal function. Such an order is negative if the disease-free equilibrium point is globally asymptotically stable (that is, the reproduction number is less than one) so that the infection converges exponentially to zero. In other words, the curves (43) and (44) can be reasonably identified with each other as it has been made in the above subsection by considering the influence of the initial conditions. In more sophisticated models involving the concourse of more subpopulations (say susceptible, immune, etc.), like those discussed in the above section, the differential equation is of higher-order than one so that the α(t) -function describing the time evolution of I(t) depends on the remaining subpopulations. This translates into the following facts:

(1) *Fact 1*: It is known that, for η=3, $\sigma_{r}=\sqrt{\frac{1}{6}}\approx 0.408$; $\frac{D_{r}}{L_{r}}=1.649$; $\frac{I\left(D_{r}\right)}{I\left(L_{r}\right)}=\frac{f\left(D_{r}\right)}{f\left(L_{r}\right)}=2.120$, [11].
(2) *Fact 2*: A modification of the relevant time instants D and L of maximum infection and previous inflection point with respect to $D_{r}$ and $L_{r}$, and the corresponding entropies as it has been discussed analytically in Section 4.1. Those parameters depend on each particular model. This also will translate, as a result, into a change of the distribution width σ related to the reference width $\sigma_{r}$ for the maximum dissipation concerns.
(3) *Fact 3*: Although the above reference values $\sigma_{r}$ and ratio $D_{r}/L_{r}$ are independent on $I_{0}$, since they are got from the log-normal distribution function, the current ones are, in general, dependent on $I_{0}$. The entropy of the current given multi-subpopulation model is given explicitly by (37), subject to (38)–(39). The time instants D and L of the respective maximum and inflection infection time instants and their values I(D) and I(L) are calculated from the first zeros of the curves $\dot{I}\left(t\right)$ and $\ddot{I}\left(t\right)$, respectively which also lead directly to their corresponding rates.
(4) *Fact 4*: The entropy of the current model might be interpreted in terms of the maximum dissipation rate by assuming a description via a log-normal distribution. However, it is easy to verify that the log-normal function is zeroed as its argument is either zero or +∞, although its profile is close, but not identical, to the solution of a first-order differential equation describing a decaying exponential infection evolution towards a disease- free equilibrium point. For this reason, and having in mind the comparison of the solution of models with more than one subpopulation (with associated differential system of order larger than one) to the log-normal distribution f(t) which is zero at zero and at infinity and which satisfies $\int_{0}^{\infty }f\left(t\right)d\left(t\right)=1$, we first normalize the infectious subpopulation of the current model in order to get a comparable entropy to the reference one associated with the log-normal function, that is, we define:
  (48) $$I_{n}\left(t\right)=\frac{I\left(t\right)}{\int_{0}^{\infty }I\left(\tau \right)d\tau };\;S_{I_{n}}\left(\eta \right)=-\int_{0}^{\infty }I_{n}\left(\tau \right)ln\;\left(I_{n}\left(\tau \right)/t^{\eta -1}\right)d\tau$$

### 4.3. Some Numerical Tests on Reference and Current Model Entropies

Now Example 2 and Example 4 are compared to the infection study of [11], by introducing the appropriate tools of normalized infection entropy (48) associated with the maximum dissipation rate for the choice η=1. Recall the basic notation $D_{r}$, $L_{r}$, D and L being the first time instants such that $\dot{I}\left(D_{r}\right)=0$, $\ddot{I}\left(L_{r}\right)=0$, $\dot{I}\left(D\right)=0$, $\ddot{I}\left(L\right)=0$ (Examples 2 and 4). One gets from (45) for η=1 and, correspondingly, $\sigma_{r}=\sqrt{1/2}$ that the parameterized reference entropy is:

(49) $$S_{I_{r}}\left(\sqrt{\frac{1}{2}},D_{r},1\right)=\left(ln\sqrt{\pi }+lnD_{r}+1\right)$$

and one gets for Example 2 that its associated normalized entropy for η=1 being un-parameterized in (D, σ) becomes from (48):

(50) $$S_{I_{n}}\left(1\right)=-\int_{0}^{\infty }I_{n}\left(t\right)lnI_{n}\left(t\right)dt$$

*Numerical experimentation with Example 2*: Note that D is the first time instant such that $\dot{I}\left(D\right)=0$ and I(D) is a relative maximum, which in practice, gives the maximum expected infectious numbers. Also, L is the first time instant such that $\ddot{I}\left(L\right)=0$. Note also that the basic model, of response being close to a log- normal function, has only an infectious subpopulation while the examples of Section 3 have more subpopulations integrated in the models. Therefore, the reasonable condition that the initial conditions of the infectious subpopulation are the one percent of the total population, we consider a total population of $N_{0}=S_{0}+I_{0}+R_{0}=1$ for Example 2 in order to get a feasible comparison. 

Thus, we perform several alternative experiments as follows:

(a) We get the values of the time instants D and L and the corresponding infection numbers I(D) and I(L), from the solution trajectory of Example 2 and its first two-time derivatives trajectories through time, as well as the normalized entropy $S_{I_{n}}\left(1\right)$ from (50). Later on, by equalizing (50) to (49), one then gets the value of $D_{rm}$ which specifies the time instant given a maximum infectious subpopulation with a maximum dissipation rate in a log normal distribution. This equalization yields:
  (51) $$D_{rm}=e^{S_{I_{n}}\left(1\right)-1}/\sqrt{\pi }$$
(b) We equalize again (49) by fixing $D_{r}=D$ in (50). Then, we get the necessary value $\sigma_{rm}$ for such an equality to hold.
(c) We define the variance with distribution function $I_{n}\left(t\right)$ and log- normal distribution resulting to be:
  (52) $$var\left(\omega \right)=\int_{0}^{\infty }\omega \left(t\right){\left(t-\int_{0}^{\infty }\omega \left(t\right)tdt\right)}^{2}dt$$
  where $\omega \left(t\right)=I_{n}\left(t\right)$ or $\omega \left(t\right)=x\left(t,D_{r},\sigma_{r}\right)$, the log-normal distribution. Then, we obtain the necessary $\sigma_{rmv}=\sigma_{rmv}\left(var\left(I_{n}\right),\;D\right)$ got from
  (53) $$var\left(I_{n}\right)=var\left(x\left(D_{r}\;=D,\sigma_{rmv}\right)\right)$$

**Figure 5:**
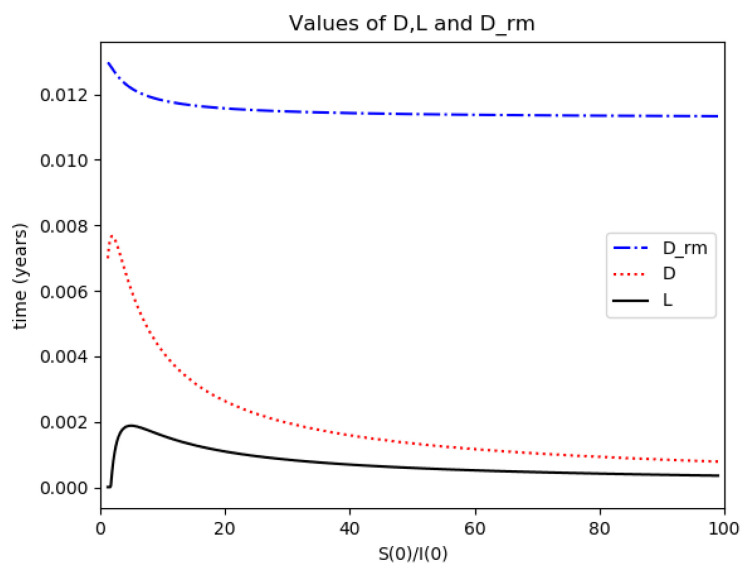
D (maximum infection time) and L (inflection point time) for Example 2.

**Figure 6:**
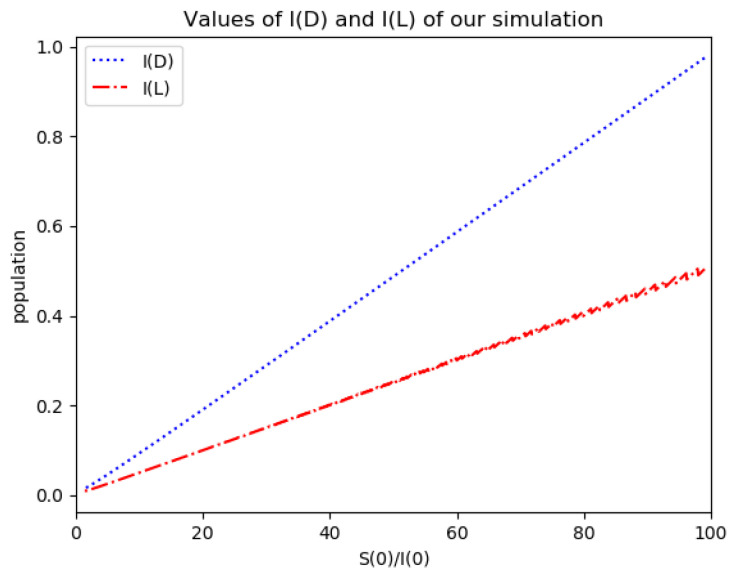
Maximum infection and inflection reached values I(D) and I(L) for Example 2.

**Figure 7:**
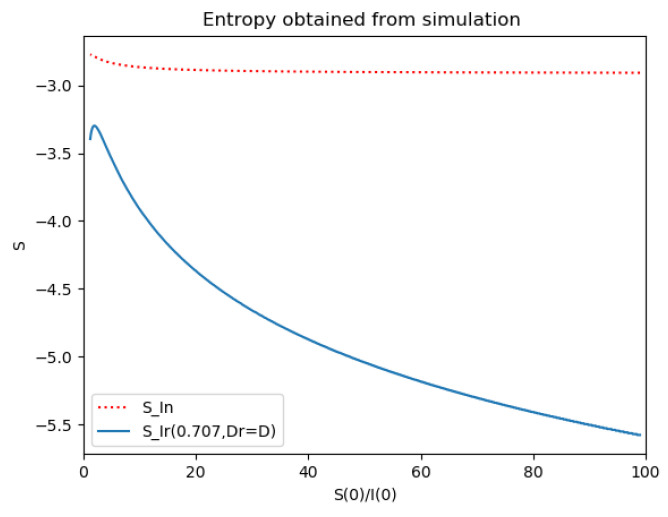
Reference and model entropies of Example 2.

**Figure 8:**
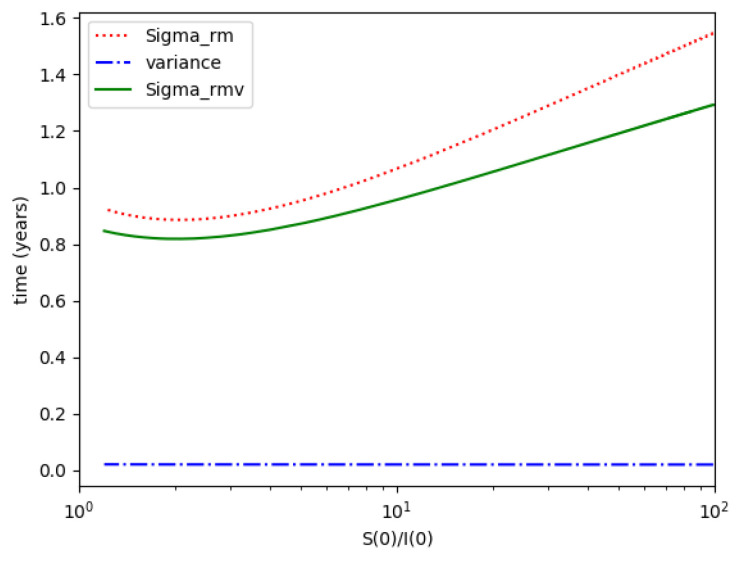
$\sigma_{rm}\;$, $\sigma_{rmv}\;$ and variance for Example 2.

One observes that, in general, $\sigma_{rmv}\neq \sigma_{r}=\frac{1}{\sqrt{2}}$ which ensures that the variance of log- normal distribution is equal to $var\left(I_{n}\right)$ for such a value of $\sigma_{rmv}$. Some numerical data on Example 2 are now compared with the log-normal distribution function. The model parameters are β=13,065 and *γ* = 50.1 year^−1^ what means that the average infectious period is *T_γ_*= 1/*γ =* 365/50.1 = 7.29 days. The initial infectious subpopulation is the one percent of the normalized total one N_0_ = 1. For those initialization, the quotient $S_{0}/I_{0}$ (percentage of initial susceptible subpopulation versus recovered subpopulation) is used to plot Figure 5, Figure 6, Figure 7 and Figure 8 whose time scale are in years. Figure 5 displays the time instants of maximum infection and inflection point versus different values of $S_{0}/R_{0}$. The values of $D_{rm}$ from (51) is also plotted. The corresponding infectious subpopulations are displayed in Figure 6. Figure 7 gives the entropies of (50) and (49). On the other hand, Figure 8 displays $\sigma_{rm}$, $\sigma_{rmv}$ and the variance of the normalized infectious $I_{n}\left(t\right)$ of (52). It is basically concluded that for the model of example 2 which has three subpopulations, the results are distinct from to those obtained from the log-normal distribution which we can recall that behave closely to the solution of a first-order differential equation involving the infectious only for initial infection being close to zero and small susceptible amounts. The above discrepancy increases as the quotient $S_{0}/I_{0}$ increases. The reason of the approximation discrepancy is that the couplings of the infectious subpopulations with the remaining ones becomes increasingly relevant to the transient responses evolution as the proportion of susceptible to infectious increases.

**Figure 9:**
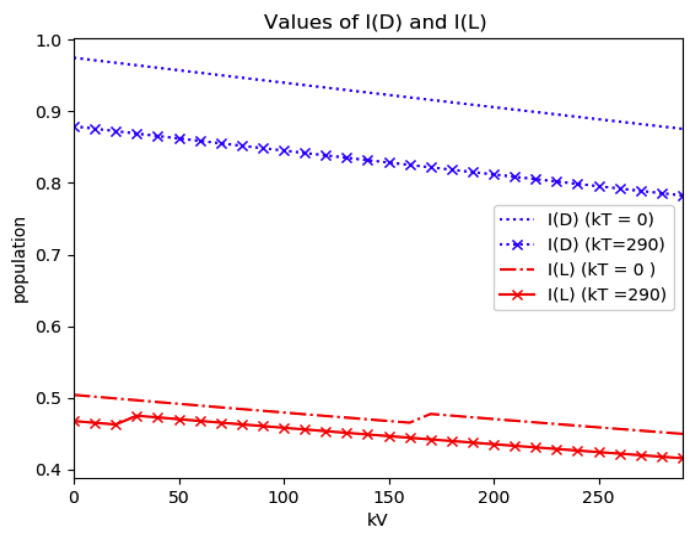
Maximum infection and its values at the inflection time instants without and with treatment control as the vaccination control effort increases for Example 4.

**Figure 10:**
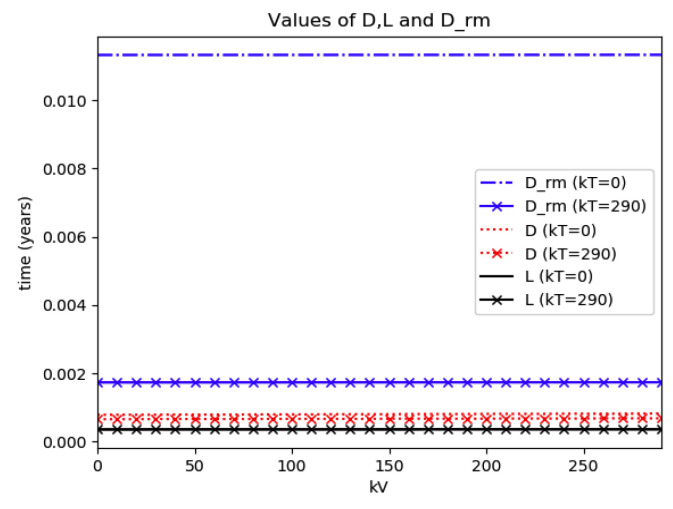
Time instants at which the maximum infection and the inflection value are reached without and with treatment control as the vaccination control effort increases for Example 4.

**Figure 11:**
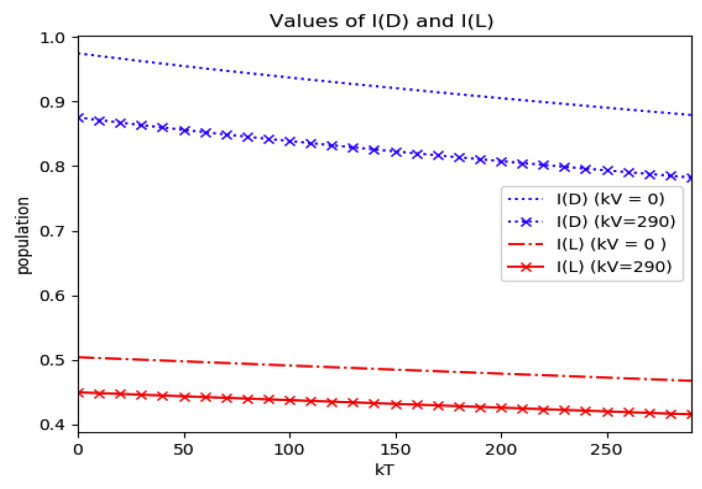
Maximum infection and its values at the inflection time instants without and with vaccination control as the treatment control gain increases for Example 4.

**Figure 12:**
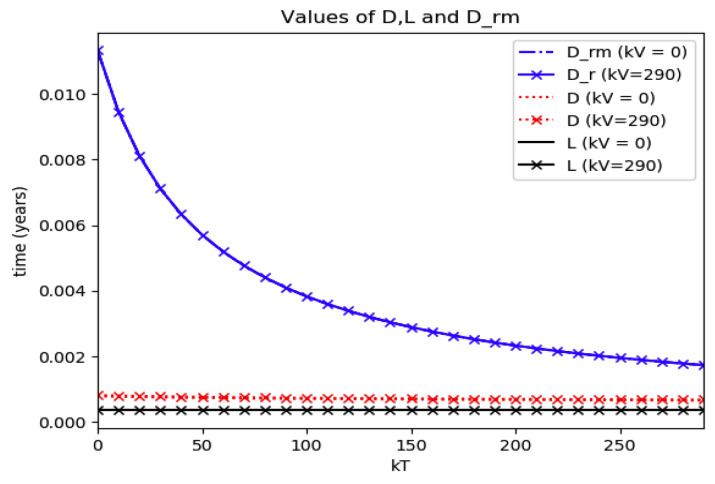
Time instants at which the maximum infection and the inflection value are reached without and with vaccination control as the treatment control effort increases for Example 4.

**Figure 13:**
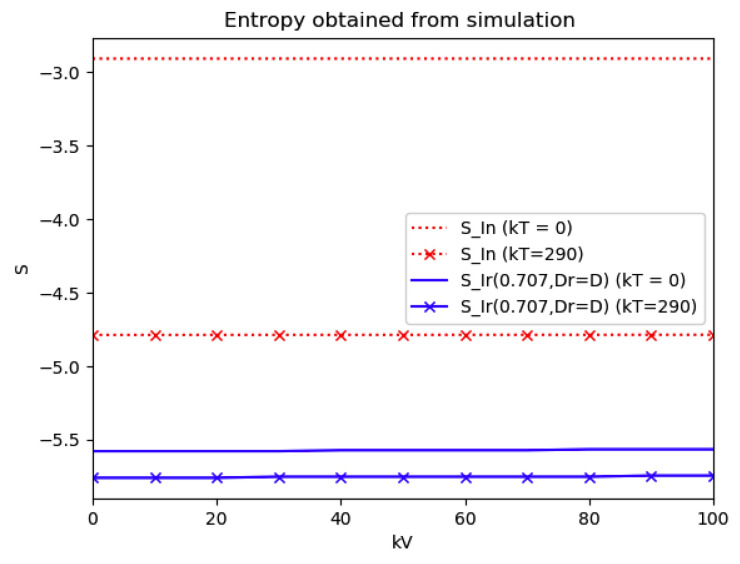
Entropies of the reference and the normalized model of Example 4 without and with treatment control as the vaccination control effort increases.

**Figure 14:**
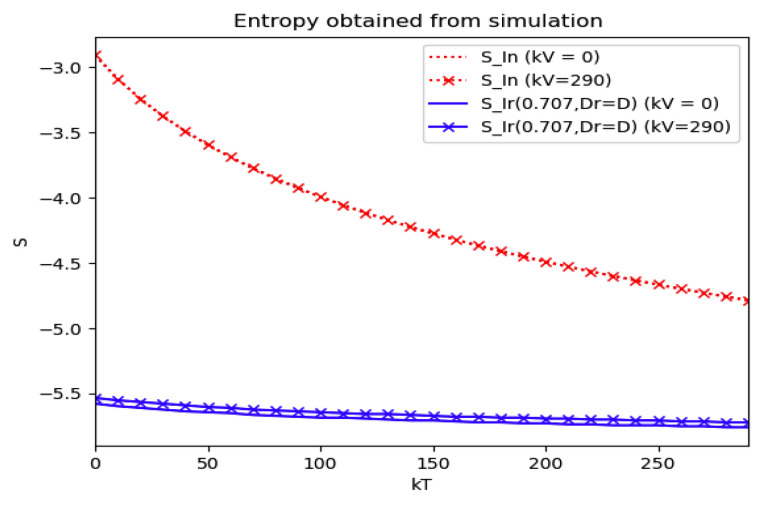
Entropies of the reference and the normalized model of Example 4 without and with vaccination control as the treatment control effort increases.

*Numerical experimentation with Example 4*: The initial values satisfy a normalization constraint $N_{0}=S_{0}+R_{0}=1$ with subpopulations *S*_0_ = 0.99, *i*_o_ = 0.01 (that, is the initial infectious subpopulation is 1% of the total one) and $R_{0}=0$ since the recovered populations is compensatory in the model in order to take into account the effects of the intervention controls. The parameters β and γ are fixed as in Example 2. In particular, Figure 9 and Figure 10 show the maximum infection and its previous value at the inflection time instant and the corresponding time instants without vaccination and with a vaccination effort rate of *k_T_* = 290 for different values of the vaccination control gain. It is basically seen that the maximum and inflection amounts decrease as the treatment control gain gives a skip from zero to an important effort as that, in parallel, the above values also decrease as the vaccination control gain increases. Figure 10 and Figure 11 describe parallel experiments where the roles of the vaccination and treatment control gains are reversed with respect to the data of Figure 9 and Figure 10. The obtained conclusions are similar. The time instants of maximum infection and the inflection value are reached without and with vaccination control as the treatment control effort increases for Example 4 are plotted in Figure 12. The corresponding entropies for those to experiments compared to the reference entropy are displayed in Figure 13 and Figure 14. Note that the entropies (48) and (50) reach negative values because of the normalization of the infection by the total infection integral contribution (48) used to evaluate the normalized entropy (50). Note that the vaccination control does not affect to the entropy as significantly as the treatment control gains since it influences less significantly to the model dynamics.

## 5. Conclusions

This paper has investigated the extensions of a first-order differential system describing the infection propagation through time to epidemic models integrating more than one subpopulation. The main involved tool has been the consideration of the coupling of inter-populations dynamics and the control intervention information through the structure of the time-varying coefficient which drives the basic differential equation model of first-order. The control of the infection along its transient to fight more efficiently against a potential initial exploding transmission from a high initial growth rate is considered relevant. Special attention has been paid throughout the manuscript to the discussion of the profiles of the transients of the infection curve in terms of the time instants of its first relative maximum towards its previous inflection time instant, so the study is mainly focused on the transient behavior characterization rather than on the steady-state equilibrium points. The time instants leading to the maximum infection and inflection numbers have been investigated via the Shannon´s information entropy for the maximum dissipation rate linked to a previous background study for a first-order differential equation describing the infection propagation. Since it is relevant to know the time instants of maximum infection and inflection as well as its numbers in order to monitor the availability of hospitalization resources, some examples related to existing epidemic models integrated by more than a subpopulation have been studied. The obtained results have been compared, both via theoretical work and also by numerical experimentation, to the background results obtained from a reference model, just involving a single infectious population, which is based on a description via a log-normal distribution which has a close profile to the solution response of a first-order differential equation. In those examples, special attention is paid to the comparisons of the maximum infection and inflection time dates for different values of initial conditions and to the entropy discrepancies related to the reference one. It can be concluded that the influence of the couplings of the dynamics of other subpopulations in the model to the infectious one is relevant to the infection evolution, especially, in the cases when the initial amounts of the susceptible are significantly large compared to the initial amounts of the infectious.
